# Episodic body size variations of early Paleozoic trilobites associated with marine redox changes

**DOI:** 10.1126/sciadv.adt7572

**Published:** 2025-05-02

**Authors:** Zhixin Sun, Fangchen Zhao, Han Zeng, Douglas H. Erwin, Maoyan Zhu

**Affiliations:** ^1^ State Key Laboratory of Palaeobiology and Stratigraphy, Nanjing Institute of Geology and Palaeontology, Chinese Academy of Sciences, Nanjing 210008, China.; ^2^ College of Earth and Planetary Sciences, University of Chinese Academy of Sciences, Beijing 100049, China.; ^3^ Department of Paleobiology, MRC-121, National Museum of Natural History, Washington, DC 20013-7012, USA.; ^4^ Santa Fe Institute, 1399 Hyde Park Road, Santa Fe, NM 87501-8943, USA.

## Abstract

Body size greatly affects how organisms interact with their environments. However, the macroevolutionary patterns of body size across many major metazoan clades and their constraining mechanisms remain elusive. A new high-resolution body size dataset covering 2435 species from 1091 genera of Cambrian and Ordovician trilobites reveals that body size evolution changes episodically, with three marked reductions in size. Such a pattern rules out a persistent Cope’s rule dynamic. Rather, we find a strong temporal link between body size changes and major fluctuations in marine redox, supporting the hypothesis that marine oxygen levels exerted a primary control on the tempo and mode of trilobite body size evolution. These further imply a dominant role for marine oxygen in early animal evolution.

## Teaser

Episodic pattern of trilobite body size evolution is shaped by ocean redox changes, rather than Cope’s rule and temperature.

## INTRODUCTION

Exploring macroevolutionary patterns and processes using the fossil record is vital to understanding how developmental drivers and ecological pressures shape biodiversity (*1*). Larger fossil datasets and advanced analytical methods have enabled precise restoration of the tree of life, and trait-based approaches enable quantification of morphological evolutionary processes (*2*–*4*).

Size is one of the most obvious organismal traits and is a key factor in determining how organisms interact with their environment (*5*–*7*), making patterns of animal body size evolution a focus of macroevolutionary research. The varied patterns of body size evolution among different clades, such as early bursts of ichthyosaurs (*8*), substantial increases among brontotheres (*9*), miniaturization of bird-related dinosaurs (*10*), and dynamic adaptations of whales (*11*), reflect the importance of body size changes in major metazoan clades. This has permitted investigation of trends in organism body size evolution, broadly divided into increases in size over geological time [Cope’s rule (*12*, *13*)], and larger sizes in cooler environments [Bergmann’s rule (*14*)]. Cope’s rule has been hypothesized to have driven patterns of body size evolution in individual clades to entire biotas (*13*, *15*, *16*), whereas Bergmann’s rule has been tested in relatively few extinct taxa (*17*). Recently, attempts at a more mechanistic understanding have emerged, with particular emphasis on oxygen as a major driver of body size, through both Cope’s rule and a temperature-size relationship (extended by Bergmann’s rule) (*13*, *16*, *18*, *19*). However, many of these studies come from fossil vertebrates. Among invertebrates, comprehensive research about body size evolution is limited to a few groups, such as brachiopods (*20*) and insects (*21*). This imbalance particularly affects our understanding of body size evolution during the early Paleozoic, a period when fluctuating environmental parameters provides an ideal opportunity to test the relative importance of oxygen levels and other mechanisms on body size.

Owing to their long duration, wide range of lifestyles, and abundant fossil record, trilobites have long been a central focus of macroevolutionary research (*22*–*24*). With body sizes ranging from ~2 mm (*Acanthopleurella stipulae*) to more than 700 mm (*Isotelus rex* or *Ogyginus forteyi*), they are an ideal clade to test the evolutionary dynamics of body size. The clade experienced two critical events: the Cambrian Explosion and subsequent Great Ordovician Biodiversification Event (GOBE) during which marine oxygen has been identified as an important control (*25*–*28*) and may have shaped the body size of trilobites. Despite long-standing interest in giant trilobites (*29*, *30*), there have been few comprehensive assessments of their body size evolution. A vaguely downward trend in trilobite body size through the Paleozoic revealed by previous studies (*13*, *31*) has low temporal resolution and was based on limited data sampling, making it difficult to assess the evolutionary dynamics of body size and evaluate their underlying causes. To exploit the macroevolutionary potential of trilobites, we compiled an extensive dataset of body size for Cambrian and Ordovician forms, encompassing 1091 genera, representing more than 90% of trilobite families during this interval (*32*). We explored the role of marine oxygen, temperature, and directional evolution in trilobite size dynamics. Our analysis underscores the long-term influence of trilobite body size evolution by marine oxygen levels, further emphasizing the important role oxygen played in shaping the evolutionary dynamics of the early metazoans.

## RESULTS

### Episodic body size evolution of early Paleozoic trilobites

**Fig. 1.**
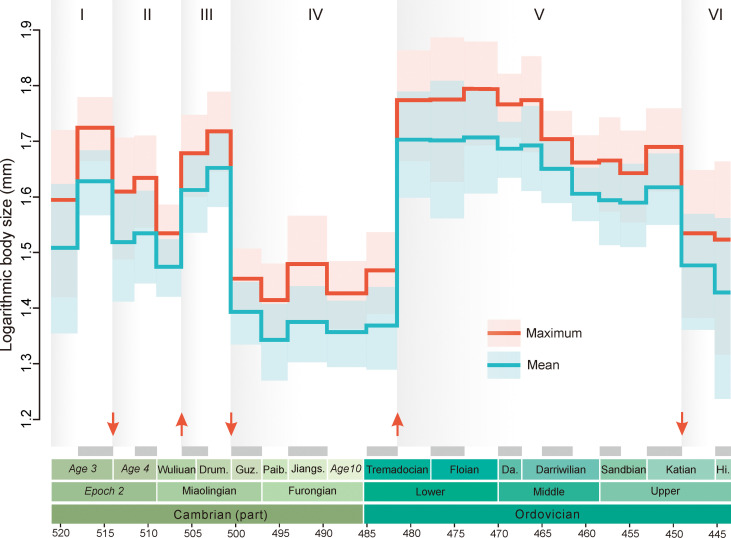
Tempo and mode in the body size evolution of Cambrian-Ordovician trilobites. Changes in maximum size (red) and mean size (blue) for each time slice, with lines and shading representing the mean value and 95% confidence intervals. Discrete episodes of body size change are marked by red arrows, and these events demarcate six distinct phases, which are labeled by the light gray shading. The temporal resolution of the dataset is shown by the alternating gray/white bars corresponding to the 24 time slices defined by the international chronostratigraphic chart and classical biostratigraphic boundaries. Drum, Drumian; Guz, Guzhangian; Paib, Paibian; Jiangs, Jiangshanian; Da, Dapingian; Hi, Hirnantian.

To explore trilobite body size changes through the Cambrian and Ordovician, we measured the complete exoskeleton length of 4732 adult trilobite specimens from the available published literature worldwide, with stratigraphic ranges in the fossil record resolved to 24 stage or substage level time slices (see Materials and Methods). By using two-sample *t* test assuming unequal variance, we tested statistical differences in size between adjacent time slices. We found that significant global trilobite body size changes (*P* < 0.05) were concentrated in five brief events in early Age 4 [~514 million years ago (Ma)], late Wuliuan (~506.5 Ma), Guzhangian (~500.5 Ma), late Tremadocian (~480 Ma), and late Katian (~450 Ma), allowing this interval to be divided into six evolutionary phases (Fig. 1 and fig. S1). Peak Cambrian body size occurred during the first 7 million years (Myr) of trilobite evolution (First Phase, Cambrian Age 3), followed by a decline during the Sinsk event and a pause through the Cambrian Age 4 and early Wuliuan (Second Phase). From the late Wuliuan to early Drumian, body sizes gradually increased, reaching a second peak in the late Drumian (Third Phase). The fourth phase began with a sharp decline (about 50%) in mean body sizes during the Guzhangian (~500.5 to 497 Ma), followed by a prolonged pause lasting over 20 Myr (Fourth Phase). This phase ended with a rapid size increase during the middle Tremadocian to a third peak in body size; thereafter, the size of trilobites leveled off after 470 Ma and progressively declined through the GOBE (Fifth Phase, ~480 to 450 Ma). On the eve of the end-Ordovician mass extinction, there was another drop in size (Sixth Phase).

**Fig. 2.**
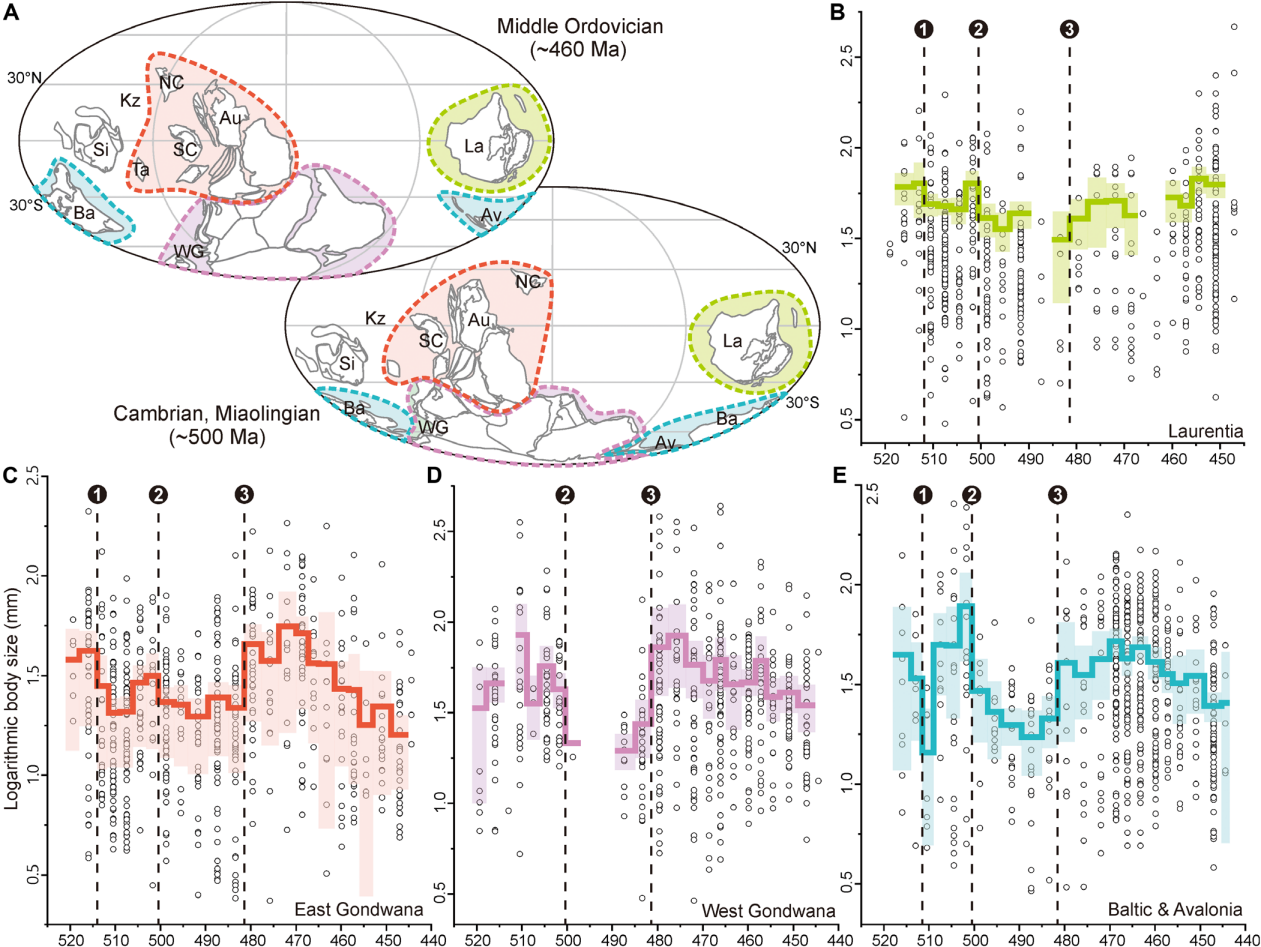
Body size evolution of Cambrian-Ordovician trilobites in four major paleogeographic regions. (**A**) Global paleogeography for the middle Cambrian and Ordovician; colors indicate the primary research regions [modified from (*74*)]. (**B** to **E**) Trilobite body size evolution in Laurentia, East Gondwana (including China and Australia), West Gondwana (Morocco, Iberia, France, Sardinia, Bohemian massif and Cordillera Oriental), Baltica, and Avalonia. Three episodes of change in body size during Cambrian Age 4, the Guzhangian, and middle Tremadocian are marked 1, 2, and 3. Abbreviations: Au, Australia; Av, Avalonia; Ba, Baltica; Kz, Kazakhstan; La, Laurentia; NC, North China; SC, South China; Si, Siberia; Ta, Tarim; WG, West Gondwana.

This episodic pattern is evident in maximum and mean sizes (Fig. 1 and fig. S2) and is not an artifact of differences in sample size across intervals (fig. S3). To evaluate whether preservational and geographic biases affected overall patterns, we analyzed trilobite size evolution across four major paleogeographic units (Laurentia, East Gondwana, Baltica and Avalonia, and West Gondwana), representing various paleoenvironmental settings from the shallow-water siliciclastic shelf to carbonate platform. The results show that the major size changes are evident in each region, particularly during Age 4, Guzhangian, and middle Tremadocian (Fig. 2), suggesting that global patterns largely mirrored regional trends. Compared with other regions, trilobites from Laurentia do show regional patterns, including no apparent increase in body size during the Tremadocian and an increase rather than decrease in the late Ordovician (Fig. 2B). The size stasis in the Tremadocian can be attributed to sampling, whereas the increase in the late Ordovician may reflect different trilobite assemblages between Laurentia and other Ordovician carbonate platforms. Correlation analyses between the global pattern and sampling sizes in different regions show that sampling biases in Laurentia, East Gondwana and Baltica did not significantly affect (*P* > 0.05) the global trilobite body size (fig. S4). The siliciclastic deposits in West Gondwana may provide more large trilobite specimens, but the linear fitting value (*R* < 0.5) indicates that the effect of this bias is low. Collectively, these suggest that trilobite body size changes were more likely influenced by global rather than regional drivers.

### No direction in early Paleozoic trilobite size evolution

To explore whether the episodic changes in body size obscures an underlying directional pattern, we observed the trend in average body size across the 24 most diverse trilobite families, covering >70% of the species in our database. Most well-sampled trilobite families (*n* = 20) exhibit no significant size changes through time (*R*^2^ < 0.1), including the dominant Asaphidae and gigantic Paradoxididae (fig. S5). This finding does not support a widespread Cope’s rule pattern at the family level. Despite some overlap, the body size distribution differs between trilobite families, especially in well-sampled families (fig. S6). These size changes are more likely concentrated among families rather than within families. Thus, we use the family as the appropriate level for assessing body size evolution.

**Fig. 3.**
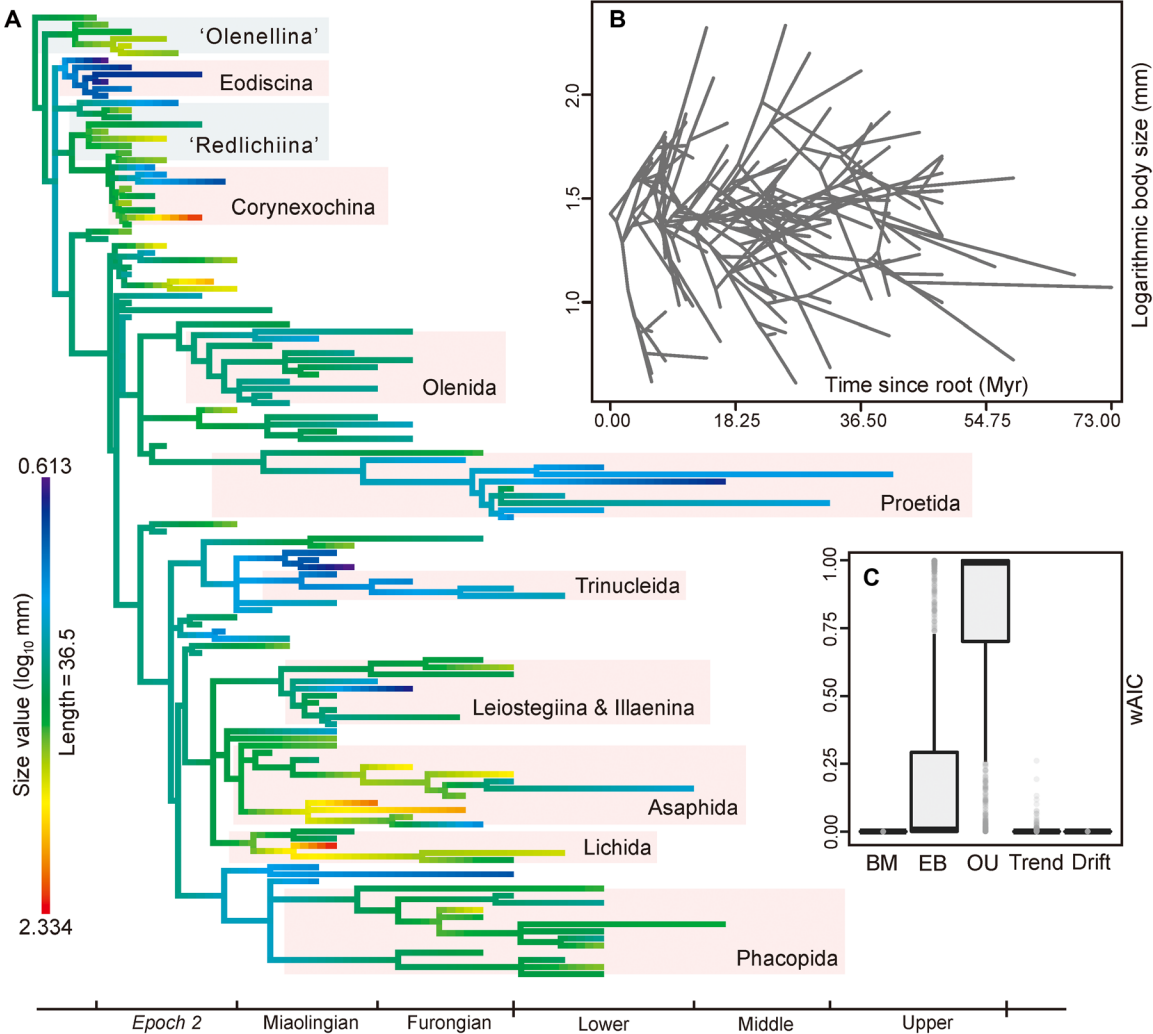
Phylogenetic-based family-level body size evolution in Cambrian-Ordovician trilobites. (**A**) Ancestral state reconstruction of body size with trait values figured as color range, showing that families with faster rates (rapid changes in color) arose independently in multiple lineages. The underlying phylogenetic framework was synthesized from previous phylogenetic analyses supplemented by traditional taxonomy where required (for details, see the Supplementary Materials). (**B**) Evolutionary traitgram of trilobite body size; values are from the analysis shown in (A). (**C**) Model fitting results expressed as Akaike weights for five different evolutionary models each with 1000 iterations. BM, Brownian motion; EB, early burst; OU, Ornstein-Uhlenbeck.

To visualize the scale and directionality of body size evolution among trilobite families, we assembled an informal trilobite supertree and plotted ancestral body size onto the family-level phylogeny (Fig. 3A and figs. S9 and S10). This calculation is based on the maximum-likelihood estimations of a synthetic tree derived from previous phylogenetic views (see Materials and Methods). The heatmap of body size shows that extreme-size families with faster rates (i.e., rapid changes in color) occur independently in multiple lineages, a pattern confirmed by traitgrams (Fig. 3B and figs. S11 and S12). This suggests that most trilobite families were near the average size, with some clades independently evolving to larger or smaller size at different ages. There is no support for an overall trend in body size evolution. As a further test for evolutionary trends, we fitted five different models of continuous trait macroevolution using mean standard length and assessed the explanatory power of each model with Akaike information criterion (AICc) and their corresponding Akaike weights (see Materials and Methods). The Ornstein-Uhlenbeck (OU) model is strongly preferred over other models, with the lowest AICc score of 66.352 and the mean AICc weight of 0.789 (median = 0.992) (Fig. 2C and fig. S13). This model describes constrained evolution around macroevolutionary “optima” or adaptive zones (*33*). The Early Burst (EB) model, which shows the high evolutionary rates in the early phase of a clade (*34*), is also supported, especially when the OU model is excluded (figs. S13 and S14), suggesting that trilobites reached the maximum size range in the early Cambrian (Fig. 2B). By contrast, no preference is indicated for other models such as Trend and Drift, which assume directional change in trait values over time (*8*, *35*). Removing the questionable miniaturized clade Eodiscina (fig. S8) from the dataset did not change these result (figs. S13, B and D, and S14, B and D). Meanwhile, removing the OU model that may be difficult to interpret did not lead to higher support for models representing directed evolution (figs. S13, C and D, and S14, C and D). Overall, our analyses reveal no preferential direction in trilobite body size through the Cambrian and Ordovician periods, thus rejecting a widespread Cope’s rule effect in trilobites of this age.

## DISCUSSION

### Marine redox control the tempo of the trilobite size evolution

**Fig. 4.**
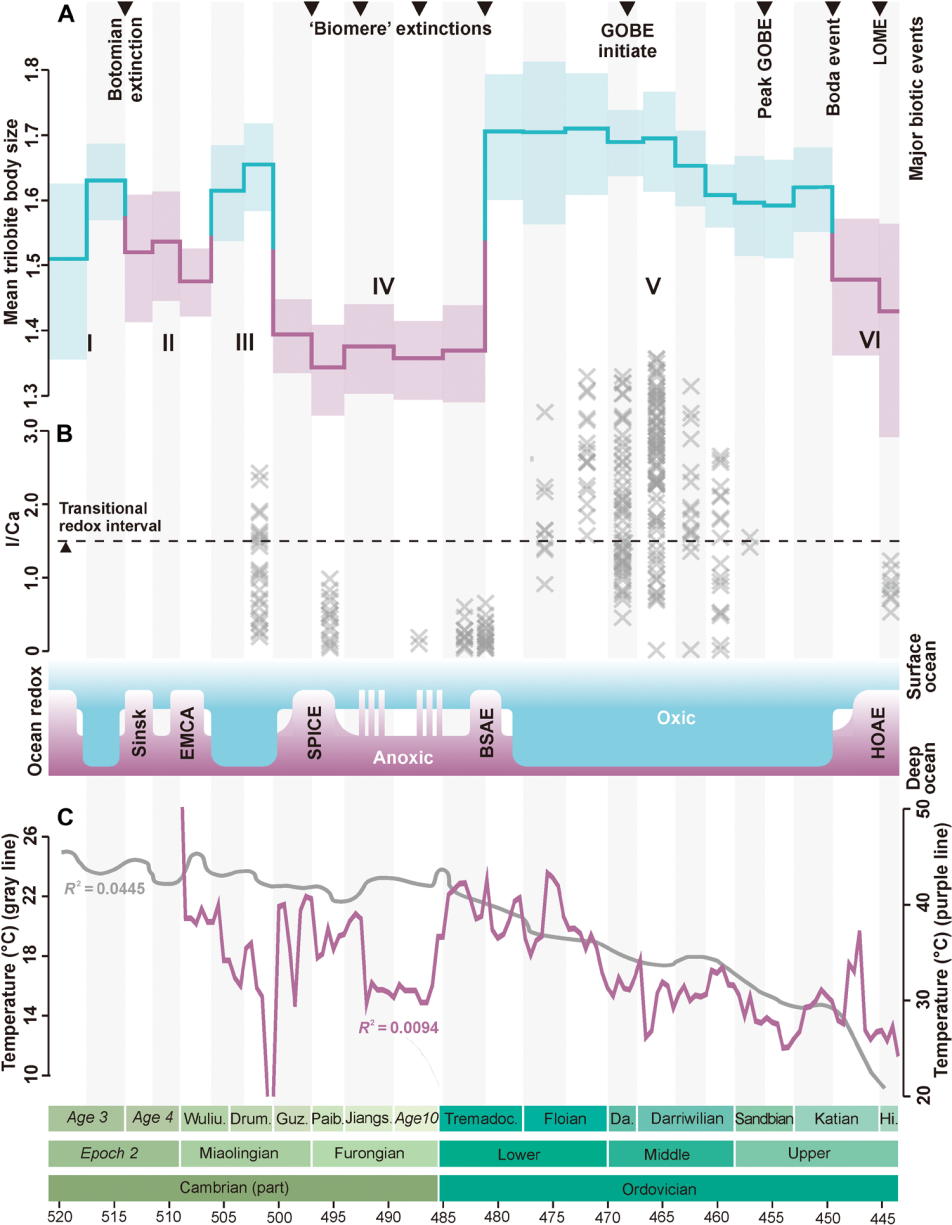
Cambrian and Ordovician trilobite body size and their correlation to the inferred oxygen levels and temperature. (**A**) Body size and major biotic events, six phases (I to VI) shown by the alternating blue/purple lines and light areas. (**B**) Ocean redox conditions and widespread anoxic intervals, showing strong links between trilobite body size and marine redox changes [based on (*25*, *27*, *36*, *38*, *40*, *42*, *44*)]; blue and purple indicate oxic and anoxic states, respectively; carbonate I/Ca values from (*28*) and (*41*); data greater than 4.0 from Floian to early Darriwilian are not shown. (**C**) Temperature curves are based on bulk rock (purple line) and skeletal material (gray line) δ18O datasets from (*75*) and (*76*), respectively. EMCA, “Early–Middle Cambrian” anoxia; SPICE, Steptoean positive carbon isotope excursion; BSAE, base Stairsian Anoxia Event; HOAE, Hirnantian Oceanic Anoxic Event. For abbreviations in the chronostratigraphic chart, see Fig. 1. Data are from dataset S16.

On the basis of these results, we explored possible environmental effects on changes in body size. Previous studies have invoked many factors as potential drivers of body size changes, with an important role for oxygen (*13*, *16*, *18*, *19*). Although some details are unclear, geochemical proxies provide evidence for marked variations under oceanic redox conditions, especially three widespread marine anoxic episodes during the Cambrian-Ordovician periods (Fig. 4, A and B). Oceans became progressively oxygenated in Cambrian Age 3 (*27*, *36*, *37*), followed by a widespread anoxic event at ~514 Ma (*38*), coinciding with the Sinsk biotic crisis. Although marine redox condition during Cambrian Age 4 to Drumian has not been fully assessed, the available data suggest that the ocean may have remained anoxic until the early Wuliuan (*39*, *40*) before becoming more oxic during the Drumian (*41*). This short-lived oxygenation was interrupted by the Steptoean positive carbon isotope excursion (SPICE), which began an episodic expansion of oxygen-depleted waters from the Guzhangian to the early Ordovician (*25*, *42*, *43*). The base Stairsian Anoxia Event (BSAE) in the late Tremadocian represents the end of a 20-Myr persistent anoxic event (*42*, *43*). Subsequently, the marine oxygenation continued to expand (*26*, *28*) until the Hirnantian Oceanic Anoxic Event (HOAE) in late Katian (*44*–*46*).

There is a notable correlation between our trilobite size data and fluctuations in marine redox (Fig. 4, A and B), especially the well-known anoxic events of this interval. The reductions in trilobite size at the end of the first phase correspond with the Sinsk event, and the 8-Myr miniaturization period (second phase) after 514 Ma coincided with sustained marine anoxia. The miniaturization from the Guzhangian to early Tremadocian (fourth phase) is consistent with the SPICE and subsequent episodic anoxic events, and termination of a persistent anoxic event (BSAE) in late Tremadocian may have triggered the most pronounced increase in trilobite size. From the late Tremadocian, trilobite size remained stable for nearly 30 Myr (fifth phase), coinciding with the expansion of marine oxygenation. Further miniaturization of trilobites in the late Katian, which again coincides with the global HOAE anoxic event, and the miniaturization phenomenon probably continued into the Devonian (*31*). In contrast, although global cooling may have triggered the extraordinary GOBE (*47*, *48*), estimates of early Paleozoic temperature changes showed little correlation with trilobite body size fluctuations (*R*^2^ < 0.1; Fig. 4C).

In short, trilobite body size changes during the Cambrian-Ordovician periods correspond closely with marine redox fluctuations. Given oxygen availability as a potential limit on body size (*16*, *18*, *49*), it is reasonable to assume that marine redox have influenced, if not directly controlled, trilobite body size changes across the world. The results here strongly support a model in which oxygen levels constrained body size evolution, which has been less understood in marine metazoans than in terrestrial clades (*18*). This pattern is consistent with the metabolic scaling of metabolism, in which quarter-power scaling reflects resource distribution requirements within organisms (*6*, *7*). Furthermore, our analysis provides an independent line of evidence to support the hypothesis that oxygen was an important driver for early animal evolution more generally and may have influenced body size evolution of other clades as well (*27*, *36*, *37*).

It is worth noting that, during both SPICE and HOAE, taxonomic diversity lagged body size reductions during declines in oxygen levels (*44*–*46*, *50*). This implies that the initial expansion of marine anoxia did not directly affect taxonomic diversity but affected the body size of trilobites, given that large trilobites were the most oxygen-hungry predators (*51*). For example, the predominant large middle Cambrian trilobites, Paradoxididae and Dorypygidae, became extinct during the Guzhangian before SPICE. Similarly, the Asaphidae, the most abundant giant Ordovician trilobite family, underwent a decline and ultimately disappeared in the late Kaitian Stage before the HOAE.

The Late Cenozoic Icehouse triggered extensive size increases in terrestrial vertebrates and marine invertebrates (*52*, *53*), but our results do not support such a connection during the Ordovician Icehouse. Given that Ordovician atmospheric oxygen levels were probably much lower than those of the Cenozoic ([e.g., (*54*)], one possibility is that marine oxygen availability was a more influential factor in limiting the size of trilobites during the early Paleozoic than temperature. In other words, the control of temperature on body size likely emerged only when atmospheric oxygen levels increased beyond a threshold or when ocean anoxic events became less frequent.

### Trilobite body size and extinction

It has long been noted that the larger-bodied taxa experienced greater extinction rates during biotic crises (*55*–*59*), but the universality of this rule has been questioned (*60*). As abundance decreases and longevity increases with increased body size, larger-bodied animals may simply be more susceptible during biotic crises. Our data support the preferential extinction of larger-bodied taxa, consistent with metabolic scaling. Metabolic rate per unit body mass declines with size. Thus, although the oxygen demand of larger bodied trilobites is expected to be relatively less than smaller-bodied forms, their total oxygen requirements would have been greater and may have been too high during anoxic episodes. Some insight into this problem may be provided by recognition that this size selectivity tends to precede the primary extinction phase. In other words, there is a decrease in mean size of trilobites with larger body size before their extinction. Similar size reduction early in these events has also been observed in late Permian gastropods (*61*) where selective extinctions predate the mass extinction by 3 to 8 Myr and may be overlooked by research that focuses on the mass extinction horizon. Such a decoupling between size-related disappearances and peak extinction during biodiversity crises provides a possible explanation for why size-selective extinction has not been more broadly recognized in other clades. Changes in abundance might also be linked to decreases in body size (*62*). Moreover, body size appears to be more sensitive than biodiversity to environmental challenges, suggesting that it may be an early warning signal of environmental crises. More attention to the miniaturization of extant animals may be warranted when assessing effects of current climate change (*59*).

## MATERIALS AND METHODS

### Trilobite body size dataset

We measured complete exoskeleton length for 4732 trilobite specimens from published fossils (up to 21 May 2024), building a database including their measured value, classifications at the species, genus, family, and order levels, maximum and minimum chronostratigraphic ages, and occurrences (datasets S1, S5, and S6). Body size was represented by sagittal length of the adult (holaspid period) in articulated specimens. For a given species, we measured as many specimens as possible to get average, maximum, and minimum values. Many trilobites continue to increase their pygidial segments after sexual maturity (*63*), so if the largest known articulated specimen has fewer pygidial segments than the largest pygidium, it obviously represents an early holaspis. In this case, we use larger cranidium or pygidium to extrapolate the maximum size of this species. Unlike the maximum value, the real minimum value is difficult to estimate unless the smallest measured specimens are known to be the youngest holaspids (the long accepted but never proven onset of sexual maturity in trilobites). Thus, we have not interpreted changes in the “minimum value,” which represent more precisely the size of the smallest known articulated specimen. To maximize geographic, stratigraphic, and taxonomic representation, we have objectively examined most available publications, including the original literature of generic names, which are published mainly in English but also in Chinese, Russian, German, French, Italian, and Czech (dataset S1). Most of the well-preserved trilobite species recorded in our dataset were derived from Laurentia (686 species), South China (460 species), West Gondwana (435 species), Baltica (404 species), Avalonia (267 species), Siberia (158 species), and Australia (118 species); the remaining quarter of data comes from a variety of geographical fauna from other tectonic units, such as Central Asian Orogeny Belt, Middle East, North China, Tarim, and Antarctica. Accordingly, these data reflect global patterns, rather than sampling differences between different regions (Fig. 2 and fig. S4). The body size dataset contains 2435 trilobite species from 1091 genera in 152 families—representing most of the Cambrian and Ordovician families (*32*). The remaining taxa are only known from cranidia and/or pygidia, which cannot be used to estimate total exoskeleton length. Family-level classification of Trilobita follows Jell and Adrain (*32*). On the basis of a recent work [see references in (*22*)], the Agnostina is excluded from Trilobita. Body sizes were log transformed prior to analysis.

To obtain high temporal resolution, we divided the Cambrian and Ordovician into 24 time slices with an average duration of 3 Myr (dataset S2). The basic divisions come from the most recent Global Chronostratigraphic Scale (*64*, *65*). Because the duration of individual stages is unequal, we use “stage slices” (*65*) to generate intervals of more nearly equal duration. Consequently, we subdivided most Ordovician stages except for the shorter duration Dapingian and Hirnantian (less than 3 Myr). The subdivisions of the Darriwilian and Sandbian follow the commonly used scheme (Dw1-3 and Sa1-2), whereas the Tremadocian, Floian, and Katian are subdivided by the first appearance datum (FAD) of *Araneograptus murrayi* (graptolite), *Oepikodus evae* (conodont), and *Diplacanthograptus complanatus* (graptolite) separately, which are the presumed most reliable global index fossils (dataset S2) (*65*). The proposed Cambrian substages (*66*) offer a useful model for dividing the two longer stages of the early Cambrian (Stages 3 and 4). In addition, we use two cosmopolitan agnostoids, *Ptychagnostus praecurrens* and *P. punctuosus* (*64*, *66*), to divide the Cambrian Wuliuan and Drumian into three slices. We manually time binned the measured data for all specimens based on biostratigraphic information from the original papers. We follow the global Cambrian trilobite biozone correlation chart used by Geyer (*67*) and used a biostratigraphic correlation chart for the Ordovician from Goldman *et al.* (*65*), which combines index fossils from carbonate and clastic lithofacies.

### Phylogenetic body size macroevolutionary analysis

There is no comprehensive phylogenetic analysis of Cambrian and Ordovician trilobites, but a previous work, especially Paterson *et al.* (*22*), provides a basis for assembling family-level phylogenetic relationships. The informal supertree used in this research covers 136 early Paleozoic trilobite families (figs. S7 and S8 and dataset S3) and was built from a manual synthesis of previous phylogenetic results. First, we establish a backbone tree using a comprehensive trilobite Bayesian tree of Paterson *et al.* (*22*). This backbone tree reflects relationships between the major Cambrian trilobite orders or superfamilies such as Olenellina, Eodiscina, Emuelloidea, Redlichioidea (including Corynexochina), Ellipsocephaloidea, Olenida, Leiostegiina (including Illaenina), and Asaphida. Then, we integrated some focused analyses [see examples in ref. (*68*)] into the backbone tree according to the sister relationships, and these intraordinal trees contain clades that overlap with the backbone tree. This expands the range of backbone tree and links the major Ordovician clades such as Proetida and Trinucleida with their Cambrian relatives. For groups that never appear in comprehensive phylogenetic analysis (such as Phacopida, Lichida, and Odontopleurida), we locate them according to traditional qualitative descriptions (see fig. S8 and caption for details).

On the basis of this phylogenetic framework, we used maximum-likelihood approaches (Brownian motion) to explore the evolution of family-level body size. We used the “timePaleoPhy” function in the “paleotree” R package (*69*) to time calibrate the phylogenetic tree and estimate the ancestral character states of body size using the “ace” function of the “ape” R package (*70*). Then, we used the “contMap” and “phenogram” functions from the “phytools” package to map body mass changes across the phylogeny (*71*), plotting as a heatmap and a projection of the phylogenetic tree in a space defined by body mass (on the *y* axis) and time (on the *x* axis), respectively (dataset S7). To assess tendencies in body size evolution, we used the “fitContinuous” function from the “geiger” package to test the fit of different evolutionary models to our results using a maximum-likelihood approach (*72*). We tested the following models: Brownian motion (*73*), OU (*33*), EB (*34*), Trend (*35*), and Drift (*8*), all of which are considered to have macroevolutionary relevance. We evaluated model fit by means of the AICc and Akaike weights for 1000 iterations, which represent the conditional probability for each of the tested models (datasets S8 to S15). Lowest AICc scores and largest Akaike weights indicate that the model fits the data better than any other tested model (figs. S13 and S14). The affinity of the Eodiscina is unresolved (fig. S8) (*22*), so the same model analysis was performed on the body size dataset with Eodiscina removed to compare the results (figs. S13, B and D, and S14, B and D). The OU model that is commonly favored at the level of generations may not biologically meaningful on this ~3-Myr timescale, so we performed the same analysis without this model (figs. S13, C and D, and S14, C and D).

## Supplementary Materials

### The PDF file includes:

Figs. S1 to S14 Legends for datasets S1 to S16 References

### Other Supplementary Material for this manuscript includes the following:

Datasets S1 to S16

## References

[1] D. Jablonski, Micro-and macroevolution: Scale and hierarchy in evolutionary biology and paleobiology. Paleobiology 26, 15–52 (2000).

[2] B. Deline, J. M. Greenwood, J. W. Clark, M. N. Puttick, K. J. Peterson, P. C. J. Donoghue, Evolution of metazoan morphological disparity. Proc. Natl. Acad. Sci. U.S.A. 115, E8909–E8918 (2018). 30181261 10.1073/pnas.1810575115 PMC6156614

[3] J.-X. Fan, S.-Z. Shen, D. H. Erwin, P. M. Sadler, N. M. Leod, Q.-M. Cheng, X.-D. Hou, J. Yang, X.-D. Wang, Y. Wang, H. Zhang, X. Chen, G.-X. Li, Y.-C. Zhang, Y.-K. Shi, D.-X. Yuan, Q. Chen, L.-N. Zhang, C. Li, Y.-Y. Zhao, A high-resolution summary of Cambrian to early Triassic marine invertebrate biodiversity. Science 367, 272–277 (2020). 31949075 10.1126/science.aax4953

[4] M. J. Benton, P. C. J. Donoghue, Paleontological evidence to date the tree of life. Mol. Biol. Evol. 24, 26–53 (2007). 17047029 10.1093/molbev/msl150

[5] R. H. Peters, *The Ecological Implications of Body Size* (Cambridge Univ. Press, 1983).

[6] G. B. West, J. H. Brown, B. J. Enquist, A general model for the origin of allometric scaling laws in biology. Science 276, 122–126 (1997). 9082983 10.1126/science.276.5309.122

[7] J. H. Brown, J. F. Gillooly, A. P. Allen, V. M. Savage, G. B. West, Toward a metabolic theory of ecology. Ecology 85, 1771–1789 (2004).

[8] P. M. Sander, E. M. Griebeler, N. Klein, J. V. Juarbe, T. Wintrich, L. J. Revell, L. Schmitz, Early giant reveals faster evolution of large body size in ichthyosaurs than in cetaceans. Science 374, eabf5787 (2021). 34941418 10.1126/science.abf5787

[9] O. Sanisidro, M. C. Mihlbachler, J. L. Cantalapiedra, A macroevolutionary pathway to megaherbivory. Science 380, 616–618 (2023). 37167399 10.1126/science.ade1833

[10] R. B. J. Benson, N. E. Campione, M. T. Carrano, P. D. Mannion, C. Sullivan, P. Upchurch, D. C. Evans, Rates of dinosaur body mass evolution indicate 170 million years of sustained ecological innovation on the avian stem lineage. PLOS Biol. 12, e1001853 (2014). 24802911 10.1371/journal.pbio.1001853 PMC4011683

[11] G. Burin, T. Park, T. D. James, G. J. Slater, N. Cooper, The dynamic adaptive landscape of cetacean body size. Curr. Biol. 33, 1787–1794.e3 (2023). 36990088 10.1016/j.cub.2023.03.014

[12] E. D. Cope, *The Primary Factors of Organic Evolution* (Open Court Publishing Company, 1904).

[13] N. A. Heim, M. L. Knope, E. K. Schaal, S. C. Wang, J. L. Payne, Cope's rule in the evolution of marine animals. Science 347, 867–870 (2015). 25700517 10.1126/science.1260065

[14] C. Bergmann, Ueber die Verhältnisse der Wärmeökonomie der Thierezuihrer Grösse. Gottinger Studien. 3, 595–708 (1847).

[15] J. Alroy, Cope's rule and the dynamics of body mass evolution in North American fossil mammals. Science 280, 731–734 (1998). 9563948 10.1126/science.280.5364.731

[16] J. L. Payne, A. G. Boyer, J. H. Brown, S. Finnegan, M. Kowalewski, R. A. Krause Jr., S. Kathleen Lyons, C. R. McClain, D. W. McShea, P. M. Novack-Gottshall, F. A. Smith, J. A. Stempien, S. C. Wang, Two-phase increase in the maximum size of life over 3.5 billion years reflects biological innovation and environmental opportunity. Proc. Natl. Acad. Sci. U.S.A. 106, 24–27 (2009). 19106296 10.1073/pnas.0806314106 PMC2607246

[17] G. Hunt, K. Roy, Climate change, body size evolution, and Cope's Rule in deep-sea ostracodes. Proc. Natl. Acad. Sci. U.S.A. 103, 1347–1352 (2006). 16432187 10.1073/pnas.0510550103 PMC1360587

[18] J. L. Payne, C. R. McClain, A. G. Boyer, J. H. Brown, S. Finnegan, M. Kowalewski, R. A. Krause Jr., S. Kathleen Lyons, D. W. McShea, P. M. Novack-Gottshall, F. A. Smith, P. Spaeth, J. A. Stempien, S. C. Wang, The evolutionary consequences of oxygenic photosynthesis: A body size perspective. Photosynth. Res. 107, 37–57 (2011). 20821265 10.1007/s11120-010-9593-1

[19] W. C. E. P. Verberk, D. Atkinson, K. N. Hoefnagel, A. G. Hirst, C. R. Horne, H. Siepel, Shrinking body sizes in response to warming: Explanations for the temperature-size rule with special emphasis on the role of oxygen. Biol. Rev. Camb. Philos. Soc. 96, 247–268 (2021). 32959989 10.1111/brv.12653 PMC7821163

[20] Z. Zhang, M. Augustin, J. L. Payne, Phanerozoic trends in brachiopod body size from synoptic data. Paleobiology 41, 491–501 (2015).

[21] M. E. Clapham, J. A. Karr, Environmental and biotic controls on the evolutionary history of insect body size. Proc. Natl. Acad. Sci. U.S.A. 109, 10927–10930 (2012). 22665762 10.1073/pnas.1204026109 PMC3390886

[22] J. R. Paterson, G. D. Edgecombe, M. S. Y. Lee, Trilobite evolutionary rates constrain the duration of the Cambrian explosion. Proc. Natl. Acad. Sci. U.S.A. 116, 4394–4399 (2019). 30782836 10.1073/pnas.1819366116 PMC6410820

[23] M. Foote, Contributions of individual taxa to overall morphological disparity. Paleobiology 19, 403–419 (1993).

[24] M. J. Hopkins, R. To, Long-term clade-wide shifts in trilobite segment number and allocation during the Palaeozoic. Proc. R. Soc. London Ser. B 289, 20221765 (2022). 10.1098/rspb.2022.1765 PMC9768642 36541173

[25] B. C. Gill, T. W. Lyons, S. A. Young, L. R. Kump, A. H. Knoll, M. R. Saltzman, Geochemical evidence for widespread euxinia in the Later Cambrian ocean. Nature 469, 80–83 (2011). 21209662 10.1038/nature09700

[26] C. T. Edwards, M. R. Saltzman, D. L. Royer, D. A. Fike, Oxygenation as a driver of the Great Ordovician Biodiversification Event. Nat. Geosci. 10, 925–929 (2017).

[27] T.-C. He, M.-Y. Zhu, B. J. W. Mills, P. M. Wynn, A. Y. Zhuravlev, R. Tostevin, P. A. E. Pogge von Strandmann, A. Yang, S. W. Poulton, G. A. Shields, Possible links between extreme oxygen perturbations and the Cambrian radiation of animals. Nat. Geosci. 12, 468–474 (2019). 31178922 10.1038/s41561-019-0357-z PMC6548555

[28] A. Lindskog, S. A. Young, C. N. Bowman, N. P. Kozik, S. M. Newby, M. E. Eriksson, J. Pettersson, E. Molin, J. D. Owens, Oxygenation of the Baltoscandian shelf linked to Ordovician biodiversification. Nat. Geosci. 16, 1047–1053 (2023).

[29] J. C. Gutiérrez-Marco, A. A. Sá, D. C. García-Bellido, I. Rábano, M. Valério, Giant trilobites and trilobite clusters from the Ordovician of Portugal. Geology 37, 443–446 (2009).

[30] D. M. Rudkin, G. A. Young, R. J. Elias, E. P. Dobrzanski, The world's biggest trilobite—*Isotelus rex* new species from the Upper Ordovician of northern Manitoba, Canada. J. Paleontol. 77, 99–112 (2003).

[31] M. A. Bell, S. J. Braddy, Cope's rule in the Ordovician trilobite family Asaphidae (order Asaphida): Patterns across multiple most parsimonious trees. Hist. Biol. 24, 223–230 (2012).

[32] P. A. Jell, J. M. Adrain, Available generic names for trilobites. Mem. Queensl. Mus. 48, 331–553 (2003).

[33] M. A. Butler, A. A. King, Phylogenetic comparative analysis: A modeling approach for adaptive evolution. Am. Nat. 164, 683–695 (2004). 29641928 10.1086/426002

[34] L. J. Harmon, J. B. Losos, T. Jonathan Davies, R. G. Gillespie, J. L. Gittleman, W. Bryan Jennings, K. H. Kozak, M. A. McPeek, F. Moreno-Roark, T. J. Near, A. Purvis, R. E. Ricklefs, D. Schluter, J. A. Schulte II, O. Seehausen, B. L. Sidlauskas, O. Torres-Carvajal, T. W. Jason, A. Ø. Mooers, Early bursts of body size and shape evolution are rare in comparative data. Evolution 64, 2385–2396 (2010). 20455932 10.1111/j.1558-5646.2010.01025.x

[35] G. Hunt, M. T. Carrano, Models and methods for analyzing phenotypic evolution in lineages and clades. Paleontol. Soci. Pap. 16, 245–269 (2010).

[36] X. Chen, H.-F. Ling, D. Vance, G. A. Shields-Zhou, M.-Y. Zhu, S. W. Poulton, L. M. Och, S.-Y. Jiang, D. Li, C. Archer, Rise to modern levels of ocean oxygenation coincided with the Cambrian radiation of animals. Nat. Commun. 6, 7142 (2015). 25980960 10.1038/ncomms8142 PMC4479002

[37] D. Wang, H.-F. Ling, U. Struck, X.-K. Zhu, M.-Y. Zhu, T.-C. He, B. Yang, A. Gamper, G. A. Shields, Coupling of ocean redox and animal evolution during the Ediacaran-Cambrian transition. Nat. Commun. 9, 2575 (2018). 29968714 10.1038/s41467-018-04980-5 PMC6030108

[38] A. Y. Zhuravlev, R. A. Wood, Anoxia as the cause of the mid-early Cambrian (Botomian) extinction event. Geology 24, 311–314 (1996).

[39] M. L. Hough, G. A. Shields, L. Z. Evins, H. Strauss, R. A. Henderson, S. Mackenzie, A major sulphur isotope event at c. 510 Ma: A possible anoxia–Extinction–Volcanism connection during the Early–Middle Cambrian transition? Terra Nova 18, 257–263 (2006).

[40] C. Chang, W.-X. Hu, K.-J. Huang, Z.-F. Wang, X.-L. Zhang, Mass extinction coincided with expanded continental margin euxinia during the Cambrian Age 4. Geophys. Res. Lett. 50, e2023GL105560 (2023).

[41] W. Lu, A. Ridgwell, E. Thomas, D. S. Hardisty, G.-M. Luo, T. J. Algeo, M. R. Saltzman, B. C. Gill, Y. Shen, H.-F. Ling, C. T. Edwards, M. T. Whalen, X. Zhou, K. M. Gutchess, L. Jin, R. E. M. Rickaby, H. C. Jenkyns, T. W. Lyons, T. M. Lenton, L. R. Kump, Z. Lu, Late inception of a resiliently oxygenated upper ocean. Science 361, 174–177 (2018). 29853552 10.1126/science.aar5372

[42] M. R. Saltzman, C. T. Edwards, J. M. Adrain, S. R. Westrop, Persistent oceanic anoxia and elevated extinction rates separate the Cambrian and Ordovician radiations. Geology 43, 807–810 (2015).

[43] C. T. Edwards, D. A. Fike, M. R. Saltzman, W. Lu, Z. Lu, Evidence for local and global redox conditions at an Early Ordovician (Tremadocian) mass extinction. Earth Planet. Sci. Lett. 481, 125–135 (2018).

[44] M. Liu, D.-Z. Chen, L. Jiang, R. G. Stockey, D. Aseal, B. Zhang, K. Liu, X.-R. Yang, D.-T. Yan, N. J. Planavsky, Oceanic anoxia and extinction in the latest Ordovician. Earth Planet. Sci. Lett. 588, 117553 (2022).

[45] C. M. Ø. Rasmussen, T. R. A. Vandenbroucke, D. Nogues-Bravo, S. Finnegan, Was the Late Ordovician mass extinction truly exceptional? Trends Ecol. Evol. 38, 812–821 (2023). 37183151 10.1016/j.tree.2023.04.009

[46] X. Lu, G. J. Gilleaudeau, B. Kendall, Uranium isotopes in non-euxinic shale and carbonate reveal dynamic Katian marine redox conditions accompanying a decrease in biodiversity prior to the Late Ordovician Mass Extinction. Geochim. Cosmochim. Acta 364, 22–43 (2024).

[47] J. A. Trotter, I. S. Williams, C. R. Barnes, C. Lécuyer, R. S. Nicoll, Did cooling oceans trigger Ordovician biodiversification? Evidence from conodont thermometry. Science 321, 550–554 (2008). 18653889 10.1126/science.1155814

[48] A. L. Stigall, C. T. Edwards, R. L. Freeman, C. M. Ø. Rasmussen, Coordinated biotic and abiotic change during the Great Ordovician Biodiversification Event: Darriwilian assembly of early Paleozoic building blocks. Palaeogeogr. Palaeoclimatol. Palaeoecol. 530, 249–270 (2019).

[49] G. Chapelle, L. S. Peck, Polar gigantism dictated by oxygen availability. Nature 399, 114–115 (1999).

[50] B. C. Gill, T. W. Dahl, E. U. Hammarlund, M. A. LeRoy, G. W. Gordon, D. E. Canfield, A. D. Anbar, T. W. Lyons, Redox dynamics of later Cambrian oceans. Palaeogeogr. Palaeoclimatol. Palaeoecol. 581, 110623 (2021).

[51] R. A. Fortey, R. M. Owens, Feeding habits in trilobites. Palaeontology 42, 429–465 (1999).

[52] G. Hunt, S. A. Wicaksono, J. E. Brown, K. G. MacLeod, Climate-driven body-size trends in the ostracod fauna of the deep Indian Ocean. Palaeontology 53, 1255–1268 (2010).

[53] J. Clavel, H. Morlon, Accelerated body size evolution during cold climatic periods in the Cenozoic. Proc. Natl. Acad. Sci. U.S.A. 114, 4183–4188 (2017). 28373536 10.1073/pnas.1606868114 PMC5402425

[54] B. J. W. Mills, A. J. Krause, I. Jarvis, B. D. Cramer, Evolution of atmospheric O_2_ through the phanerozoic, revisited, revisited. Annu. Rev. Earth Planet. Sci. 51, 253–276 (2023).

[55] D. M. Raup, Biological extinction in Earth history. Science 231, 1528–1533 (1986). 11542058 10.1126/science.11542058

[56] J. D. Olden, Z. S. Hogan, M. J. Vander Zanden, Small fish, big fish, red fish, blue fish: Size-biased extinction risk of the world's freshwater and marine fishes. Glob. Ecol. Biogeogr. 16, 694–701 (2007).

[57] P. M. Monarrez, N. A. Heim, J. L. Payne, Mass extinctions alter extinction and origination dynamics with respect to body size. Proc. R. Soc. London Ser. B Biol. Sci. 288, 20211681 (2021). 10.1098/rspb.2021.1681 PMC8493190 34610766

[58] P. M. Monarrez, N. A. Heim, J. L. Payne, Reduced strength and increased variability of extinction selectivity during mass extinctions. R. Soc. Open Sci. 10, 230795 (2023). 37771968 10.1098/rsos.230795 PMC10523066

[59] F. A. Smith, R. E. E. Smith, S. K. Lyons, J. L. Payne, Body size downgrading of mammals over the late Quaternary. Science 360, 310–313 (2018). 29674591 10.1126/science.aao5987

[60] J. L. Payne, N. A. Heim, Body size, sampling completeness, and extinction risk in the marine fossil record. Paleobiology 46, 23–40 (2020).

[61] J. L. Payne, Evolutionary dynamics of gastropod size across the end-Permian extinction and through the Triassic recovery interval. Paleobiology 31, 269–290 (2005).

[62] P. M. Hull, S. A. F. Darroch, D. H. Erwin, Rarity in mass extinctions and the future of ecosystems. Nature 528, 345–351 (2015). 26672552 10.1038/nature16160

[63] N. C. Hughes, A. Minelli, G. Fusco, The ontogeny of trilobite segmentation: A comparative approach. Paleobiology 32, 602–627 (2006).

[64] S.-C. Peng, L. E. Babcock, P. Ahlberg, “The Cambrian Period” in *Geologic Time Scale 2020, Volume 2* (Elsevier, 2020).

[65] D. Goldman, P. M. Sadler, S. A. Leslie, M. J. Melchin, F. P. Agterberg, F. M. Gradstein, “The Ordovician Period” in *Geologic Time Scale 2020, Volume 2* (Elsevier, 2020).

[66] L. Babcock, S.-C. Peng, P. Ahlberg, X. Zhang, M.-Y. Zhu, P. Yu. Parkhaev, A model for subdividing Cambrian stages into substages. Abstract book, in *3rd International Congress on Stratigraphy* (Società Geologica Italiana, 2019), p. 142.

[67] G. Geyer, A comprehensive Cambrian correlation chart. Episodes 42, 321–332 (2019).

[68] B. S. Lieberman, T. S. Karim, Tracing the trilobite tree from the root to the tips: A model marriage of fossils and phylogeny. Arthropod Struct. Dev. 39, 111–123 (2010). 19854298 10.1016/j.asd.2009.10.004

[69] D. W. Bapst, paleotree: An R package for paleontological and phylogenetic analyses of evolution. Methods Ecol. Evol. 3, 803–807 (2012).

[70] E. Paradis, *Analysis of Phylogenetics and Evolution with R* (Springer Science & Business Media, 2011).

[71] L. J. Revell, phytools: An R package for phylogenetic comparative biology (and other things). Methods Ecol. Evol. 3, 217–223 (2012).

[72] L. J. Harmon, J. T. Weir, C. D. Brock, R. E. Glor, W. Challenger, GEIGER: Investigating evolutionary radiations. Bioinformatics 24, 129–131 (2008). 18006550 10.1093/bioinformatics/btm538

[73] J. Felsenstein, Maximum-likelihood estimation of evolutionary trees from continuous characters. Am. J. Hum. Genet. 25, 471–492 (1973). 4741844 PMC1762641

[74] T. H. Torsvik, L. R. M. Cocks, “Cambrian” in *Earth History and Palaeogeography*, T. H. Torsvik, L. R. M. Cocks, Eds. (Cambridge Univ. Press, 2017).

[75] S. L. Goldberg, T. M. Present, S. Finnegan, K. D. Bergmann, A high-resolution record of early Paleozoic climate. Proc. Natl. Acad. Sci. U.S.A. 118, e2013083118 (2021). 33526667 10.1073/pnas.2013083118 PMC8017688

[76] C. R. Scotese, H.-J. Song, B. J. W. Mills, D. G. van der Meer, Phanerozoic paleotemperatures: The earth's changing climate during the last 540 million years. Earth Sci. Rev. 215, 103503 (2021).

[77] D. A. Harper, B. Cascales-Miñana, T. Servais, Early Palaeozoic diversifications and extinctions in the marine biosphere: A continuum of change. Geol. Mag. 157, 5–21 (2020).

[78] F. A. Sundberg, G. Geyer, P. D. Kruse, L. B. McCollum, T. V. Pegel, A. Zylinska, A. Y. Zhuravlev, International correlation of the Cambrian Series 2-3, Stages 4-5 boundary interval. Australas. Palaeontol. Mem. 49, 83–124 (2016).

[79] A. Bignon, B. G. Waisfeld, N. E. Vaccari, B. D. Chatterton, Reassessment of the Order Trinucleida (Trilobita). J. System. Palaeontol. 18, 1061–1077 (2020).

[80] N. C. Hughes, Ontogeny, intraspecific variation, and systematics of the Late Cambrian trilobite *Dikelocephalus*. Smithson. Contrib. Paleobiol. 79, 1–89 (1994).

[81] R. A. Fortey, The first known complete lichakephalid trilobite, Lower Ordovician of Morocco. Mem. Assoc. Australas. Palaeontol. 42, 1–7 (2012).

[82] J. M. Adrain, Class Trilobita Walch, 1771. Zootaxa 3148, 104–109 (2011).

[83] P. A. Jell, Phylogeny of early cambrian trilobites. Spec. Pap. Palaeontol. 70, 45–57 (2003).

[84] S. Lee, D. Lee, D. K. Choi, Cambrian–Ordovician trilobite family Missisquoiidae Hupé, 1955: Systematic revision and palaeogeographical considerations based on cladistic analysis. Palaeogeogr. Palaeoclimatol. Palaeoecol. 260, 315–341 (2008).

[85] T. S. Park, J. E. Kim, S. Lee, D. K. Choi, *Mansuyia* Sun, and *Tsinania* Walcott, from the Furongian of North China and the evolution of the trilobite family Tsinaniidae. Palaeontology 57, 269–282 (2014).

[86] R. A. Fortey, R. M. Owens, The Arenig series in South Wales. Bulletin of the British Museum (Natural History). Geology 41, 69–305 (1987).

[87] B. S. Lieberman, Phylogenetic analysis of some basal Early Cambrian Trilobites, The Biogeographic origins of the Eutrilobita, and the timing of the Cambrian Radiation. J. Paleontol. 76, 692–708 (2002).

[88] J. R. Paterson, G. D. Edgecombe, The Early Cambrian Trilobite Family Emuellidae Pocock, 1970: Systematic position and revision of Australian species. J. Paleontol. 80, 496–513 (2006).

[89] A. R. Palmer, L. N. Repina, Through a glass darkly: Taxonomy, phylogeny, and biostratigraphy of the Olenellina. Univ. Kansas Paleontol. Contrib. 3, 1–35 (1993).

[90] P. A. Jell, “Introduction to the suborder Eodiscina” in *Treatise on Invertebrate Paleontology, Part O, Arthropoda 1. Trilobita, Revised. Volume 1* R. L. Kaesler, Ed. (Geological Society of America and Univ. of Kansas Press, 1997).

[91] W.-T. Zhang, Y.-H. Lu, Z.-L. Zhu, Y.-Y. Qian, H.-L. Lin, Z.-Y. Zhou, S.-G. Zhang, J.-L. Yuan, *Cambrian trilobite faunas of southwestern China* (Science Press, 1980).

[92] N. P. Suvorova, Corynexochoid trilobites and their evolutionary history. Trudy Paleontol. Inst. 103, 1–319 (1964).

[93] J. O. Ebbestad, G. E. Budd, Burlingiid trilobites from Norway, with a discussion of their affinities and relationships. Palaeontology 45, 1171–1195 (2002).

[94] G. Geyer, Exotic trilobites from the Lower–Middle Cambrian boundary interval in Morocco and their bearing on the Cambrian Series 3 lower boundary. Paläontol. Z. 89, 749–781 (2015).

[95] N. P. Suvorova, Cambrian trilobites from the eastern Siberian Platform. Part 2. Olenellidae-Granulariidae. Trudy Paleontol. Inst. 84, 1–238 (1960).

[96] T. J. Cotton, The phylogeny and systematics of blind Cambrian ptychoparioid trilobites. Palaeontology 44, 167–207 (2001).

[97] L. E. Babcock, Systematics and phylogenetics of polymeroid trilobites from the Henson Gletscher and Kap Stanton Formations (Middle Cambrian), north Greenland. Bull. Grønl. Geol. Unders. 169, 79–127 (1994).

[98] J. C. Lamsdell, P. A. Selden, Phylogenetic support for the monophyly of proetide trilobites. Lethaia 48, 375–386 (2015).

[99] L. N. Repina, V. V. Khomentovsky, I. T. Zhuravleva, A. Yu. Rozanov, *Lower Cambrian Biostratigraphy of the Sayan-Altay Folded Region* (Akademiya Nauk SSSR, Sibirskoe Otdelenie, Institut Geologii i Geofiziki, Izdatelstvo, 1964).

[100] J. M. Adrain, S. E. Peters, S. R. Westrop, The Marjuman trilobite *Cedarina* Lochman: Thoracic morphology, systematics, and new species from western Utah and eastern Nevada, USA. Zootaxa 2218, 35–58 (2009).

[101] D. S. Monti, V. A. Confalonieri, First cladistic analysis of the trilobite family Olenidae from the Furongian and Ordovician. Lethaia 52, 304–322 (2019).

[102] C. J. Bentley, J. B. Jago, Wuaniid trilobites of Australia. Mem. Assoc. Australas. Palaeontol. 30, 179–191 (2004).

[103] D. Lee, B. D. Chatterton, Protaspides of Upper Cambrian *Aphelaspis* (Ptychopariida, Trilobita) and related species with their taxonomic implications. Palaeontology 48, 1351–1375 (2005).

[104] D. Lee, B. D. Chatterton, Hystricurid trilobite larvae from the Garden City Formation (Lower Ordovician) of Idaho and their phylogenetic implications. J. Paleontol. 71, 862–877 (1997).

[105] J. M. Adrain, A synopsis of Ordovician trilobite distribution and diversity. Geol. Soc. Lond. Mem. 38, 293–332 (2013).

[106] M. C. Ebach, K. McNamara, A systematic revision of the family Harpetidae (Trilobita). Rec. West. Aust. Mus. 21, 235–267 (2002).

[107] J.-L. Yuan, W.-T. Zhang, Z.-L. Zhu, *Cambrian Stratigraphy and Trilobite Fauna in Southern and Western Marginal Parts of the Ordos Platform* (Science Press, 2016).

[108] F. A. Sundberg, Redescription of *Alokistocare subcoronatum* (Hall and Whitfield, 1877), the type species of *Alokistocare*, and the status of Alokistocaridae Resser, 1939B (Ptychopariida: Trilobita, Middle Cambrian). J. Paleontol. 73, 1126–1143 (1999).

[109] R. A. Fortey, “Classification” in *Treatise on Invertebrate Paleontology, Part O, Arthropoda 1. Trilobita, Revised. Volume 1*, R. L. Kaesler, Ed. (Geological Society of America and Univ. of Kansas Press, 1997).

[110] P. D. Lane, A. T. Thomas, A review of the trilobite Suborder Scutelluina. Spec. Pap. Palaeontol. 30, 141–160 (1983).

[111] G. D. Edgecombe, “Trilobite phylogeny and the Cambrian–Ordovician “Event”: Cladistic reappraisal” in *Extinction and Phylogeny*, M. J. Novacek, Q. D. Wheeler, Eds. (Columbia Univ. Press, 1992).

[112] R. A. Fortey, B. D. E. Chatterton, Classification of the trilobite suborder Asaphina. Palaeontology 31, 165–222 (1988).

[113] C. Lochman, The evolution of some upper cambrian and lower ordovician trilobite families. J. Paleontol. 30, 445–462 (1956).

[114] S. R. Westrop, J. D. Eoff, T. Ng, A. A. Dengler, J. M. Adrain, Classification of the Late Cambrian (Steptoean) trilobite genera *Cheilocephalus* Berkey, 1898 and *Oligometopus* Resser, 1936 from Laurentia. Can. J. Earth Sci. 45, 725–744 (2008).

[115] R. A. Fortey, Ontogeny, hypostome attachment and trilobite classification. Palaeontology 33, 529–576 (1990).

[116] L. Ramsköld, Pattern and process in the evolution of the Odontopleuridae (Trilobita). The Selenopeltinae and Ceratocephalinae. Trans. R. Soc. Edinb. Earth Sci. 82, 143–181 (1991).

[117] J. R. Pollitt, R. A. Fortey, M. A. Wills, Systematics of the trilobite families Lichidae Hawle & Corda, 1847 and Lichakephalidae Tripp, 1957: The application of Bayesian inference to morphological data. J. Syst. Palaeontol. 3, 225–241 (2005).

[118] F. R. Rasetti, Phylogeny of the Cambrian trilobite family Catillicephalidae and the ontogeny of *Welleraspis*. J. Paleontol. 28, 599–612 (1954).

[119] R. A. Fortey, Cambrian-Ordovician trilobites from the boundary beds in western Newfoundland and their phylogenetic significance. Spec. Pap. Palaeontol. 30, 179–211 (1983).

[120] B. D. Chatterton, D. J. Siveter, G. D. Edgecombe, A. S. Hunt, Larvae and relationships of the Calymenina (Trilobita). J. Paleontol. 64, 255–277 (1990).

[121] R. A. Fortey, Trilobite systematics: The last 75 years. J. Paleontol. 75, 1141–1151 (2001).

[122] J. M. Adrain, S. R. Westrop, E. Landing, R. A. Fortey, Systematics of the Ordovician trilobites *Ischyrotoma* and *Dimeropygiella*, with species from the type Ibexian area, western U.S.A. J. Paleontol. 75, 947–971 (2001).

[123] J. Moysiuk, J. B. Caron, Burgess Shale fossils shed light on the agnostid problem. Proc. R. Soc. London Ser. B 286, 20182314 (2019). 10.1098/rspb.2018.2314 PMC6367181 30963877

