# Supplementary material for: Episodic body size variations of early Paleozoic trilobites associated with marine redox changes
Source: Sci Adv. 2025 May 2;11(18):eadt7572. doi: 10.1126/sciadv.adt7572 (PMC12047424; doi:10.1126/sciadv.adt7572)
Supplement: Supplementary file 1 — Figs. S1 to S14 Legends for datasets S1 to S16 References [file sciadv.adt7572_sm.pdf]

Science Advances
AAAS

Supplementary Materials for

Episodic body size variations of early Paleozoic trilobites associated with marine redox changes

Zhixin Sun *et al.*

Corresponding author: Fangchen Zhao, fczhao@nigpas.ac.cn; Maoyan Zhu, myzhu@nigpas.ac.cn

Sci. Adv. **11**, eadt7572 (2025)
DOI: 10.1126/sciadv.adt7572

The PDF file includes:

Figs. S1 to S14
Legends for datasets S1 to S16
References

Other Supplementary Material for this manuscript includes the following:

Datasets S1 to S16

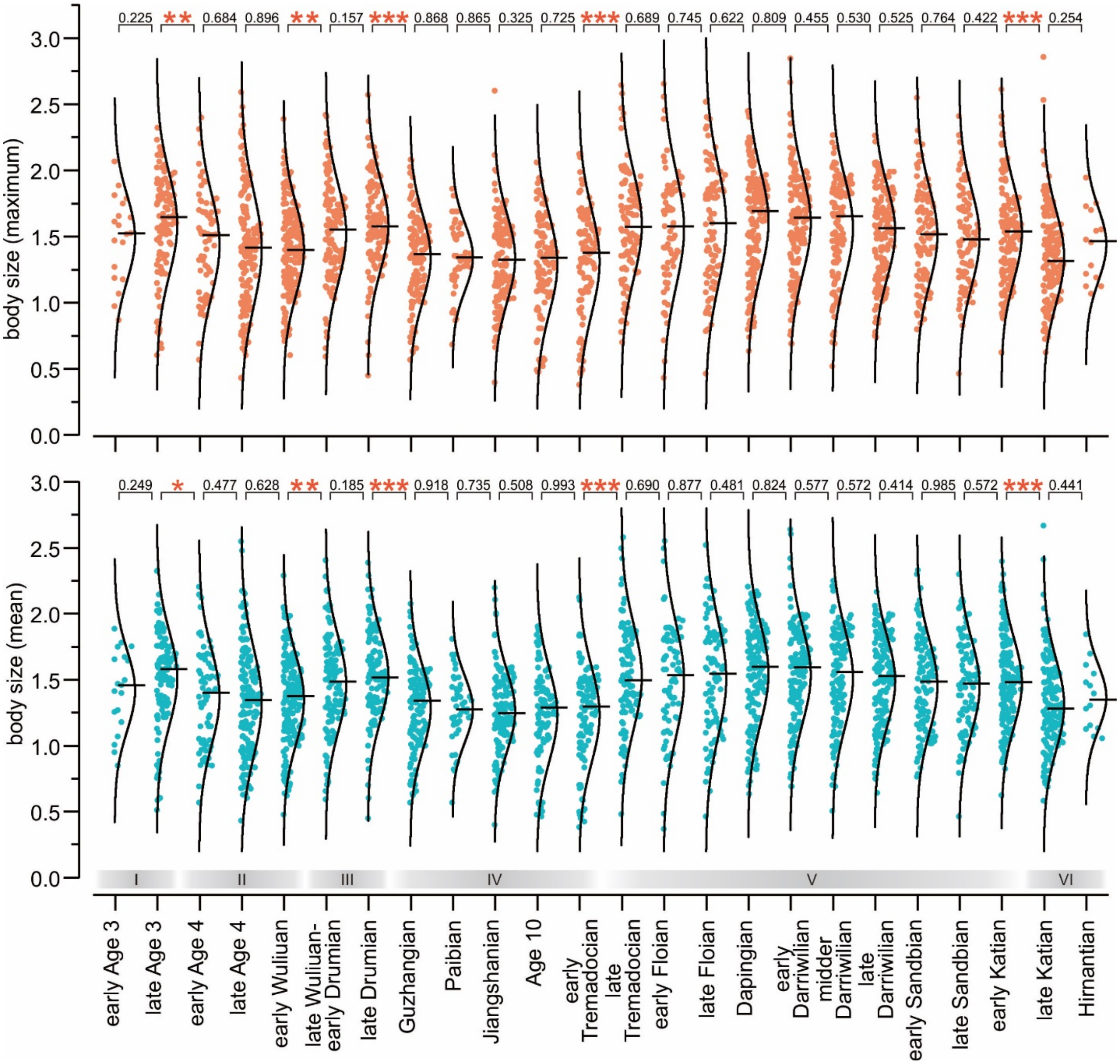

Fig. S1. The distribution of logarithmic trilobite size (mm) for each time slice, differences between the adjacent slices are shown by p-value based on a two-sample t-test assuming unequal variance. The body size evolution is divided into six phases (I-VI) according to statistically significant ($p < 0.001$ = ***, $p < 0.01$ = ***, $p < 0.05$ = *). The line represents the median of time slice. Date from Dataset S1.

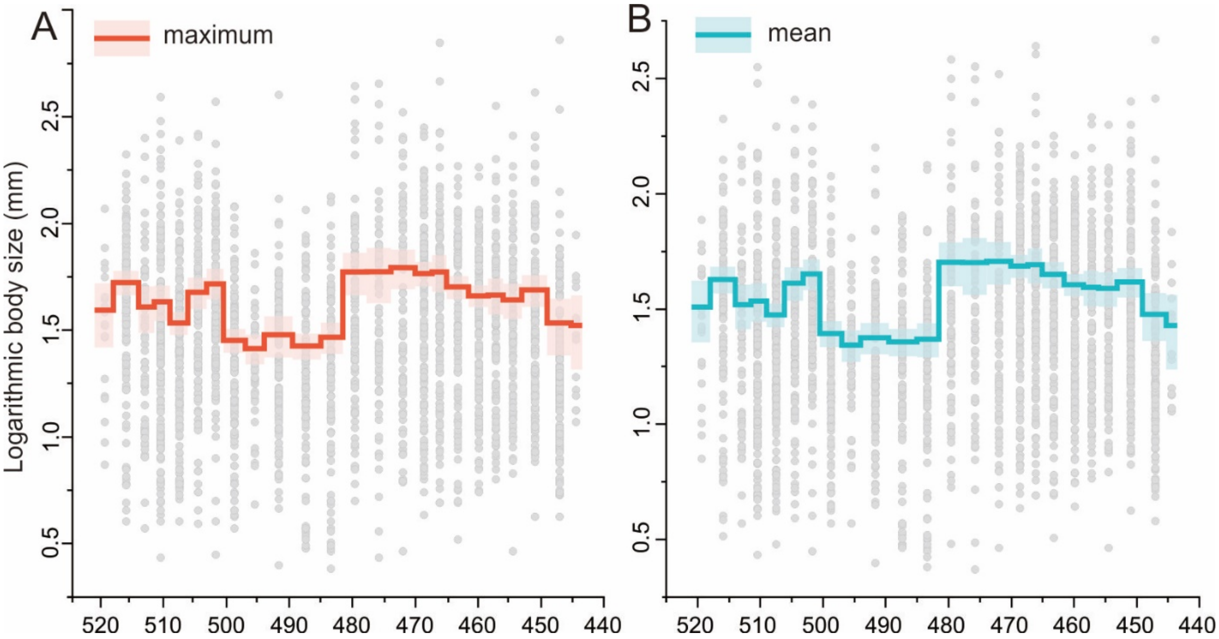

Fig. S2. The distribution of logarithmic trilobite size (mm) in maximum size (red), mean size (blue) and minimum size (gray) in each time slice, lines represent mean value, light areas represent 95% confidence intervals. Date from Dataset S1.

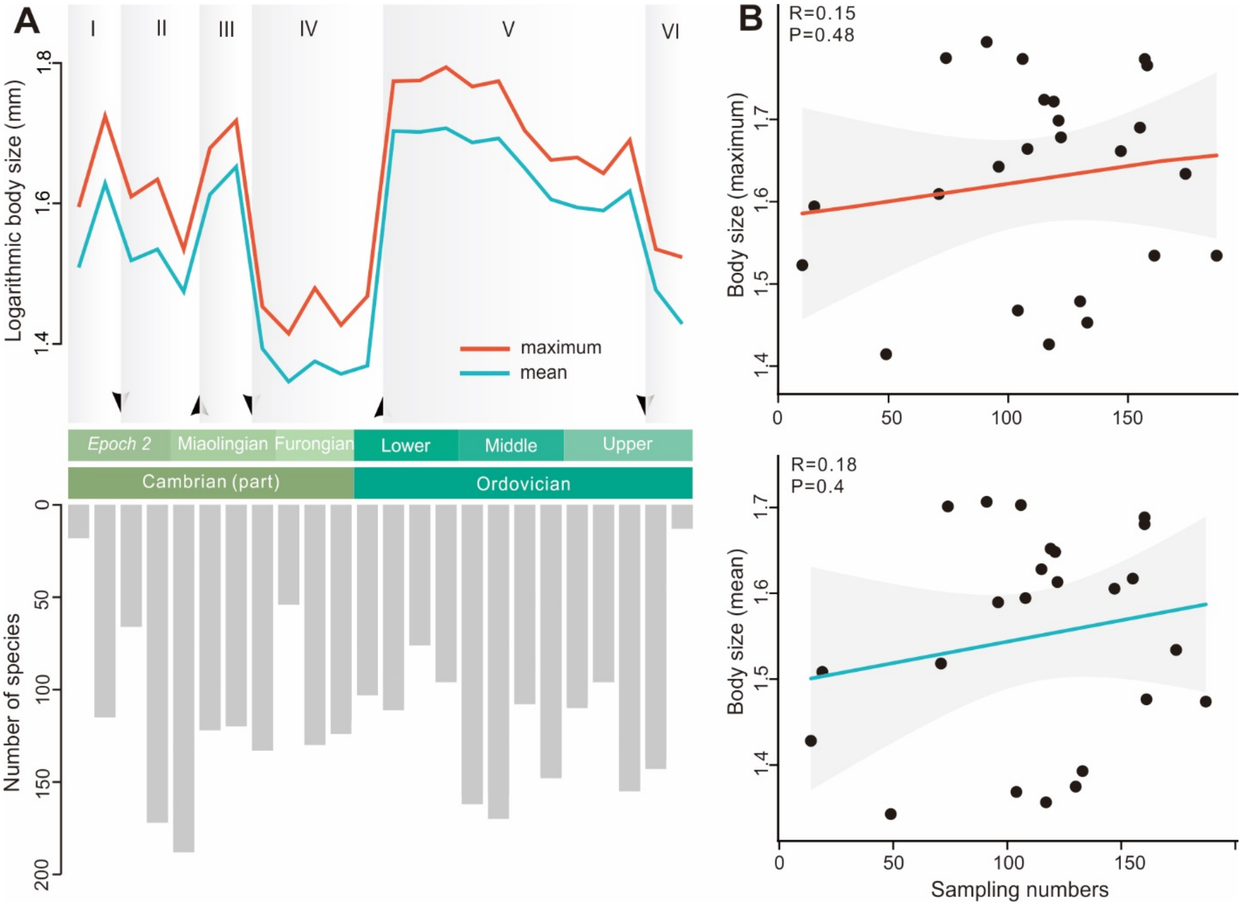

Fig. S3. The body size sampling of the Cambrian-Ordovician trilobites. (A) Globe tempo and mode in the body size evolution of the Cambrian-Ordovician trilobites. (B) Histogram of sampling numbers per time bin. The early Cambrian Age 3 and Hirnantian show obvious under-sampling, which is attributed to the low global trilobite diversity at that time, especially in the Late Ordovician (77). Samples from the early-middle Cambrian boundary, early Middle Ordovician and Sandbian-Kation are unusually rich, and the former may result from a long-term interest in studying this boundary (review in ref. 78). (C) Correlation analyses between trilobite body size (mean and maximum) and sampling numbers in each time slice, showing no significant correlation between sampling and body size ($p>0.05$, $R<0.2$). Date from Dataset S1.

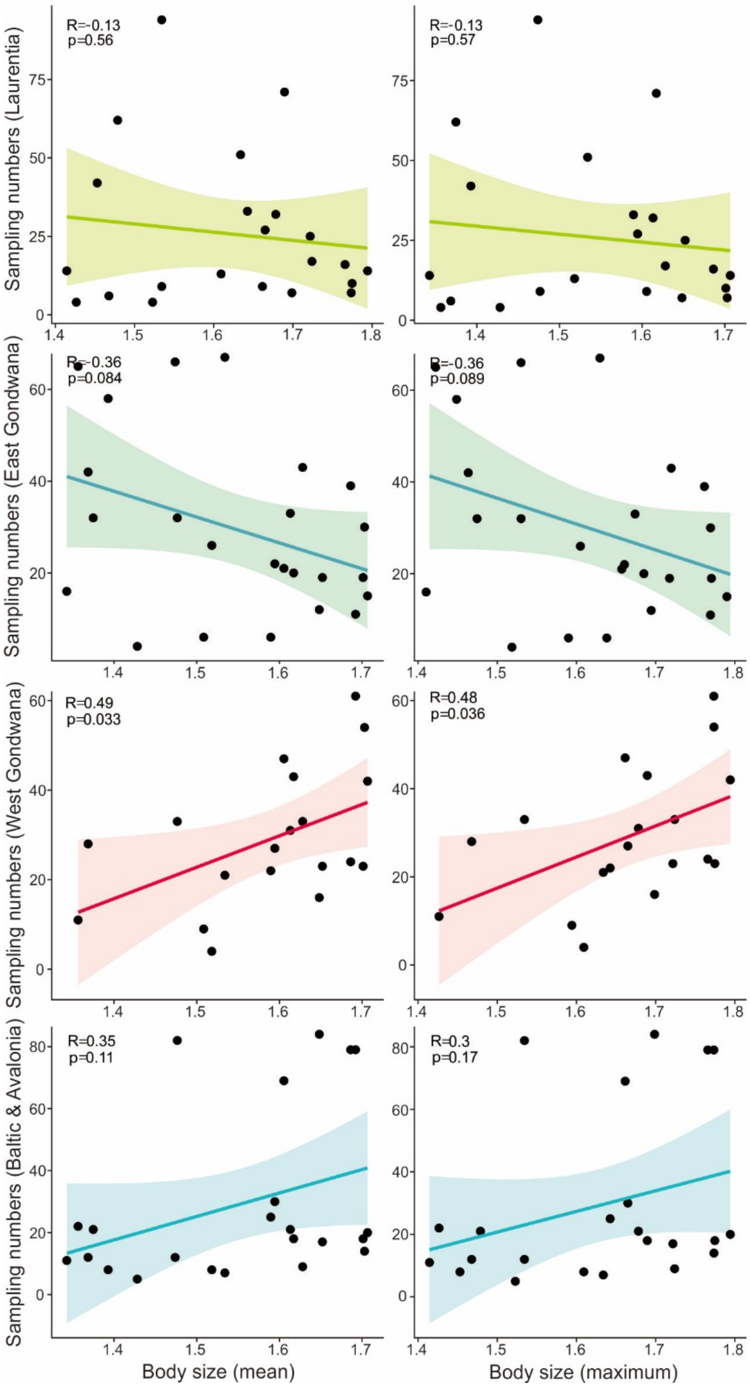

Fig. S4. Correlation analyses between global trilobite body size (left: mean; right: maximum) and sampling numbers in major research regions (Laurenia, East Gondwana, West Gondwana, Baltic and Avalonia), which reject the possibility that the changes in global body size reflect difference sampling of different regions. East Gondwana includes China and Australia, and West Gondwana includes Morocco, Iberia, France, Sardinia and Bohemian massif. Data from Dataset S1.

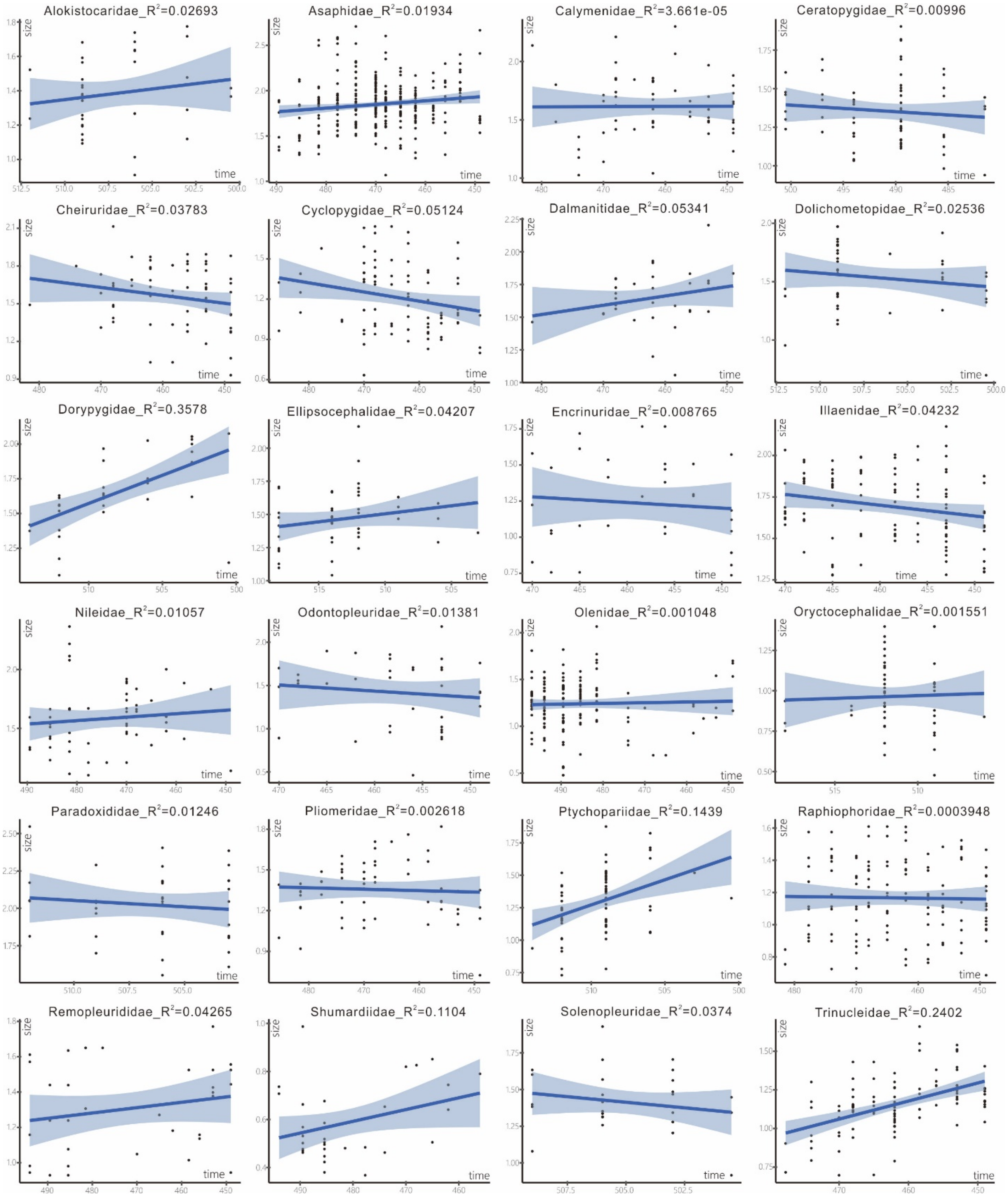

Fig. S5. Trend of average body size in major trilobite families, showing that the body size evolution of most families (R^2 <0.1, n=20) is stasis. Only Ptychopariidae, Shumardiidae, Dorypygidae, and Trinucieidae show a clear increased trend through time, especially the latter two. Considering that Trinucieidae is a specialized and miniaturized trilobite group (79), the driving mechanism of its body size evolution is worthy of further investigation in the future.

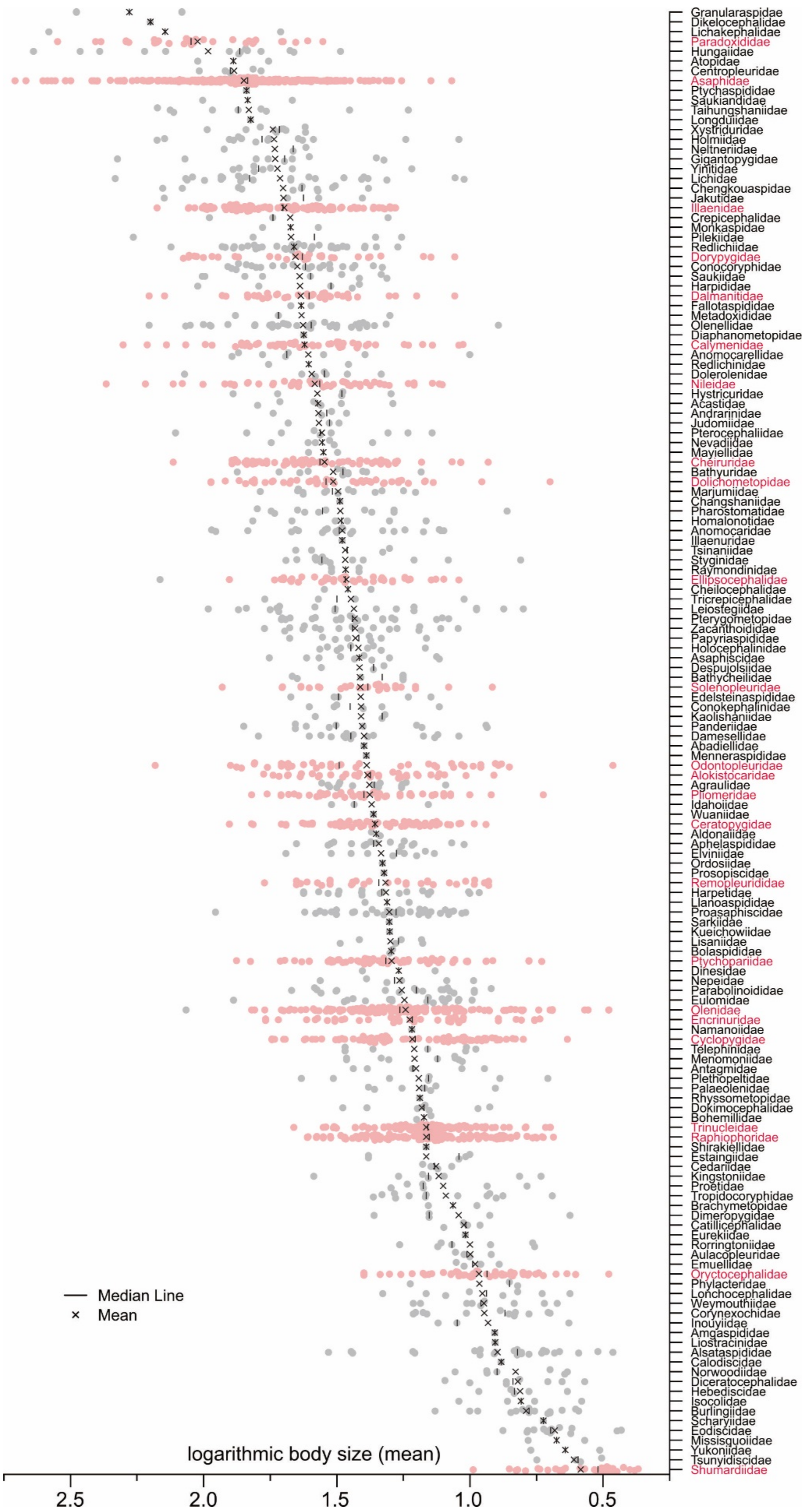

Granularaspidae
Dikelocephalidae
Lichakephalidae
Paradoxididae
Hungaiidae
Atopidae
Centropleuridae
Asaphidae
Ptychaspididae
Saukiandidae
Taihungshaniidae
Longduiidae
Xystriduridae
Holmiidae
Neltneriidae
Gigantopygidae
Yinitidae
Lichidae
Chengkouaspidae
Jakutidae
Illaenidae
Crepicephalidae
Monkaspidae
Pilekiidae
Redlichiidae
Dorypygidae
Conocoryphidae
Saukiidae
Harpididae
Dalmanitidae
Fallotaspididae
Metadoxididae
Olenellidae
Diaphanometopidae
Calymenidae
Anomocarellidae
Redlichinidae
Dolerolenidae
Nileidae
Hystricuridae
Acastidae
Andrarinidae
Judomiidae
Pterocephaliidae
Nevadiidae
Mayiellidae
Cheiruridae
Bathyuridae
Dolichometopidae
Marjumiidae
Changshaniidae
Pharostomatidae
Homalonotidae
Anomocaridae
Illaenuridae
Tsinaniidae
Styginidae
Raymondinidae
Ellipsocephalidae
Cheilocephalidae
Tricrepicephalidae
Leiostegiidae
Pterygometopidae
Zacanthoididae
Papyriaspididae
Holocephalinidae
Asaphiscidae
Despujolsiidae
Bathycheilidae
Solenopleuridae
Edelsteinaspididae
Conokephalinidae
Kaolishaniidae
Panderiidae
Damesellidae
Abadiellidae
Menneraspididae
Odontopleuridae
Alokistocaridae
Agraulidae
Pliomeridae
Idahoiidae
Wuaniidae
Ceratopygidae
Aldonaiidae
Aphelaspididae
Elviniidae
Ordosiidae
Prosopiscidae
Remopleurididae
Harpetidae
Llanoaspididae
Proasaphiscidae
Sarkiidae
Kueichowiidae
Lisaniidae
Bolaspididae
Ptychopariidae
Dinesidae
Nepeidae
Parabolinoididae
Eulomidae
Olenidae
Encrinuridae
Namanoiidae
Cyclopygidae
Telephinidae
Menomoniidae
Antagmidae
Plethopeltidae
Palaeolenidae
Rhyssometopidae
Dokimocephalidae
Bohemillidae
Trinucleidae
Raphiophoridae
Shirakiellidae
Estaingiidae
Cedariidae
Kingstoniidae
Proetidae
Tropidocoryphidae
Brachymetopidae
Dimeropygidae
Catillicephalidae
Eurekiidae
Rorringtoniidae
Aulacopleuridae
Emuellidae
Oryctocephalidae
Phylacteridae
Lonchocephalidae
Weymouthiidae
Corynexochidae
Inouyiidae
Amgaspididae
Liostracinidae
Alsataspididae
Calodiscidae
Norwoodiidae
Diceratocephalidae
Hebediscidae
Isocolidae
Burlingiidae
Scharyiidae
Eodiscidae
Missisquoiidae
Yukoniidae
Tsunyidiscidae
Shumardiidae
Median Line
Mean
logarithmic body size (mean)
2.5
2.0
1.5
1.0
0.5

Fig. S6. Distribution of average trilobite body size in family level. The well-sampled trilobite families (shown in fig. S5) are marked in red. Despite most trilobite families were near the average size, the body size distribution of different trilobite families has obvious difference, especially in the well-sampled families. In addition to the well-known Eodiscina, miniaturized trilobites also include Shumardiidae, Missisquoiidae, Scharyiidae, Brulingiidae, Isocolidae, Diceratocephalidae etc., of which Shumardiidae is the smallest trilobite group in average size. This suggests that there is an adaptive landscape that drives various polymerids to independently evolve into this size range. The Granularaspidae, Dikelocephalidae and Lichakephalidae are larger than our familiar Paradoxidiae and Asaphidae, represents the largest trilobite groups on average size. However, the data for these poorly sampled families often come from very large individuals (80, 81), which may allow their body size to be overestimated. Date from Datasets S1, 5.

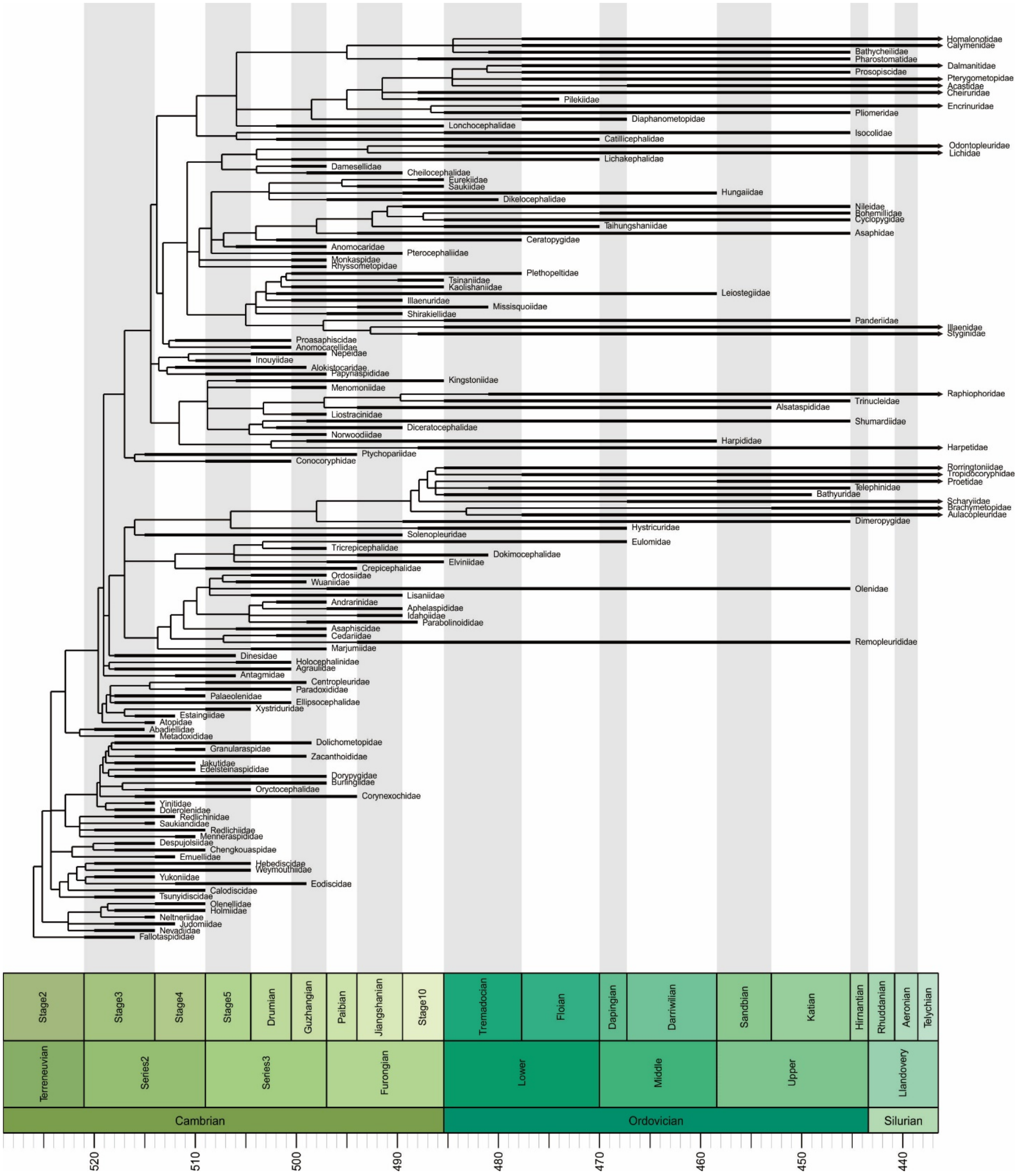

Fig. S7. Family level phylogenetic tree for Cambrian and Ordovician trilobite with geological time scale, based on a synthesis of previous work. References to families, phylogenies, and temporal occurrences are found in Fig. S8 and Datasets S3, 4.

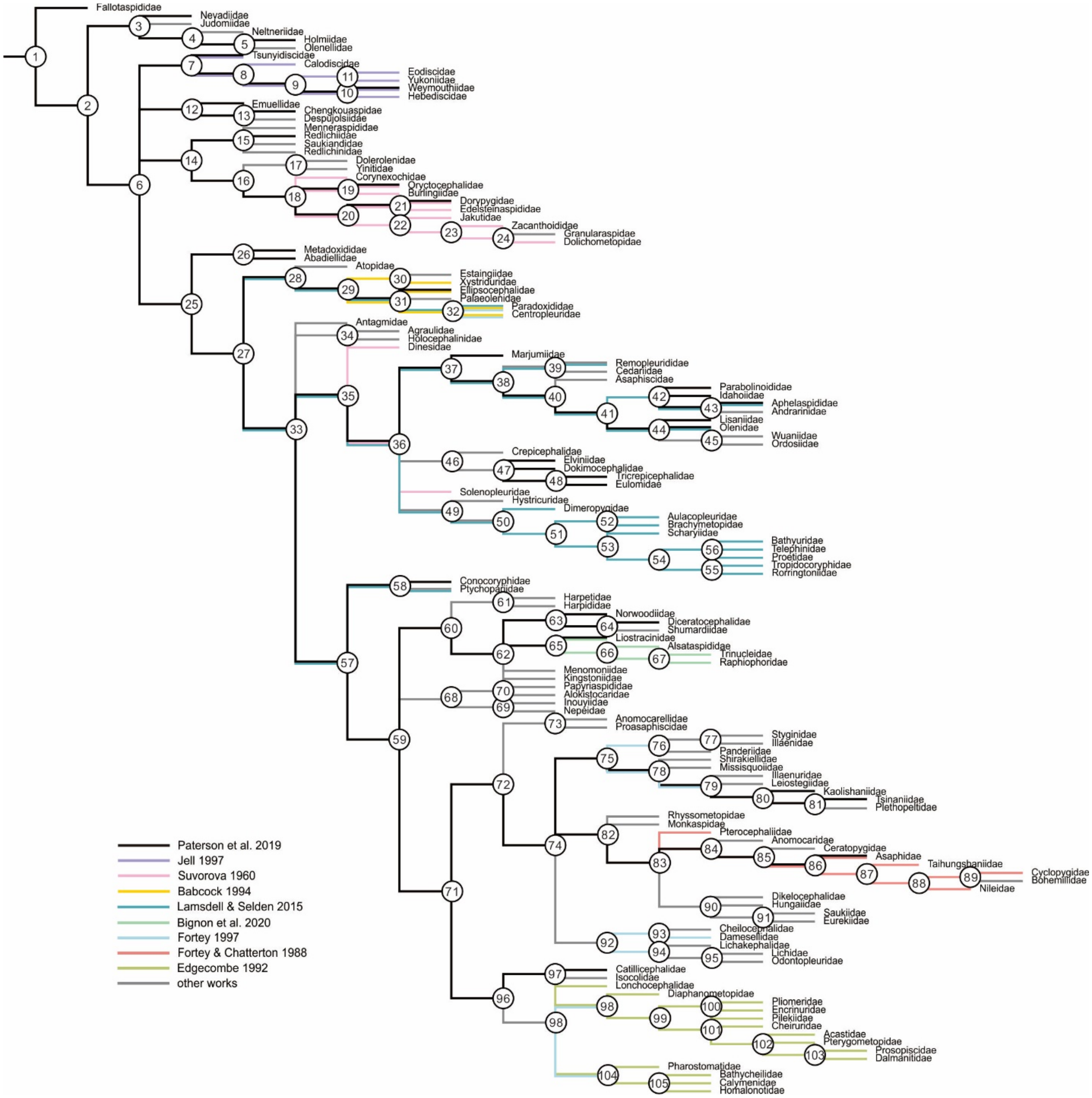

Fig. S8. The sources of the trilobite phylogenetic relationships used in this paper, and the colors show contributions from major works. Nodes 1, 2, 6, 12, 14, 16, 25–27, 37, 41, 42, 44, 46, 47, 57, 60, 62–64, 68, 71, 74, 78–82, 89, 90 from Paterson et al. (22), 60, 71, 78, 80, 82 also support by Adrain (82), Jell (83), Lee et al. (84), Park et al. (85) and Fortey (86) respectively; Node 3 from Lieberman (87) and Paterson & Edgecombe (88). Node 4: from Paterson & Edgecombe (88); Node 5 from Palmer & Repina (89); Nodes 7–11 from Jell (90); Nodes 13 and 17 from unpublished data; Nodes 15, 29, 31 from Zhang et al. (91); Nodes 18–23 from Suvorova (92); Node 19 from Ebbestad & Budd (93); Node 24 from Geyer (94); Nodes 27, 59, 72 from Jell (83); Node 28 from: Suvorova (95) and Cotton (96); Nodes 29–32 from Babcock (97); Node 33 from Suvorova (95), Jell (83), Adrain (82) and Lamsdell & Selden (98); Nodes 34 and 58 from Cotton (96); Node 35 from Repina et al. (99); Nodes 36, 38, and 76 from Adrain (82); Node 39 from Adrain et al. (100); Nodes 40 and 43 from Monti & Confalonieri (101); Node 45 from Bentley &

Jago (102); Node 48 from Lee & Chatterton (103); Node 49 from Lee & Chatterton (104); Nodes 50–56 from Lamsdell & Selden (98), 51 and 56 also support by Adrain (105) and Fortey & Owens (86) respectively; Node 61 from Ebach & McNamara (106); Nodes 65–67 from Bignon et al. (79); Nodes 69 and 73 from Yuan et al. (107); Node 70 from Sundberg (108); Nodes 75 and 76 from Fortey (109); Node 77 from Lane & Thomas (110); Nodes 83–103 from Edgecombe (111); Nodes 86–88 from Fortey & Chatterton (112); Node 91 from Lochman (113); Nodes 92 and 93 from Westrop et al. (114), 92 also support by Fortey (115); Node 94 from Ramsköld (116); Node 95 from Pollitt et al. (117); Node 96 from Rasetti (118); Node 97 from Fortey (119); Node 98 from Chatterton et al. (120); Node 104 from Fortey (121); Node 105 from Edgecombe (111) and Adrain et al. (122).

To build this tree, we firstly use Paterson et al.'s tree (22) to determine the relationships among major trilobite groups (order or superfamily level) (black lines). This backbone tree places Olenellina as the most basal group of the trilobite tree, followed by Eodiscina, Emuelloidea, and Redlichioidea (including the Corynexochina). In the ptychopariids, the group represented by *Olenus* (Order Olenida) is the most basal, and the tiny trilobites (Diceratocephalidae + Liostracinidae + Norwoodiidae) form a sister group with the clades of Leiostegiina (including Illaenina) and Asaphida. A series of ptychopariid-like redlichioids, such as Ellipsocephaloidea, are located at the base of ptychopariids.

According to the backbone tree, we assess family-level affinities within major trilobite groups based on more focused studies. For example, we constructed phylogenetic trees of Eodiscina, Olenellina, Ellipsocephaloidea, and Asaphida, based on Jell (83), Paterson & Edgecombe (88), Babcock (97) and Fortey & Chatterton (112) respectively. In the same way, we added Proetida to the Olenida clade based on outgroups in Lamsdell & Selden's tree (98); added Trinucleida to the tiny trilobite clade based on the position of Liostracinidae in Bignon et al.'s tree (79) (see corresponding lines); use Catillicephalidae to associate Phacopida with Asaphida (118, 120). For Lichida and Odontopleurida, we associate them with Damesellidae according to the traditional view (109, 115, 121). At the same time, many small adjustments were inevitable, such as we moved the position of the Dikelokephaloidea, Ceratopygidae and Corynexochidae in the Paterson et al.'s tree to maintain the monophyly of traditional Asaphida and Corynexochina.

The affinity of the Eodiscina is unresolved for a long time. Some works believe that this group originated from Redlichiina, or more precisely Ellipsocephaloidea (83). The recent phylogenic tree inferred from tip-dated Bayesian analyses shows that Eodiscina is nested in Olenellina, or is the sister group of Redlichiina (22). However, the parsimony tree based on the same matrix put this clade in ptychopariids (22. Fig S3). Furtherly, given the possible relationship between Eodiscina and agnostids (83, 90, 109), Eodiscina may even be excluded from trilobita (123). Due to their small proportion in the sample (8% in late Age 3 to Age 4, 5% in early Wuliuan), suspected affinity of the Eodiscina has little effect on the time-based body size evolution. However, the uncertain affinity of this miniaturized group may affect the conclusions of trilobite body size evolution based on phylogeny. Thus, we will discuss the model evaluation and simulation of trilobite body size in with or without Eodiscina respectively (figs. S13, 14).

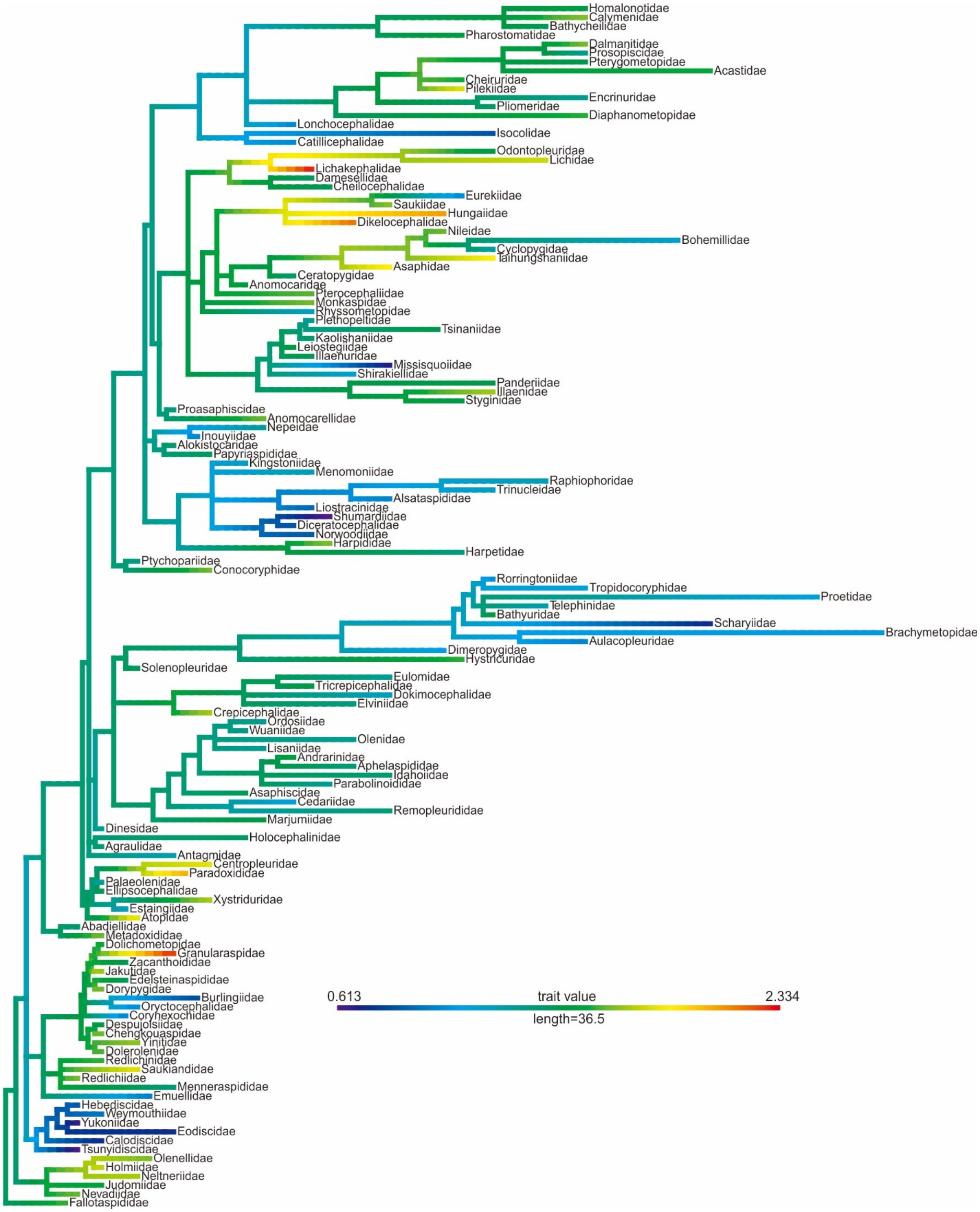

Fig. S9. Ancestral state reconstruction of mean body size (mm) for major Cambrian and Ordovician trilobite families. Changes in color reflect the evolutionary rates of body size, showing some clades independently evolving to larger or smaller size at different ages. Date from Datasets S3-5.

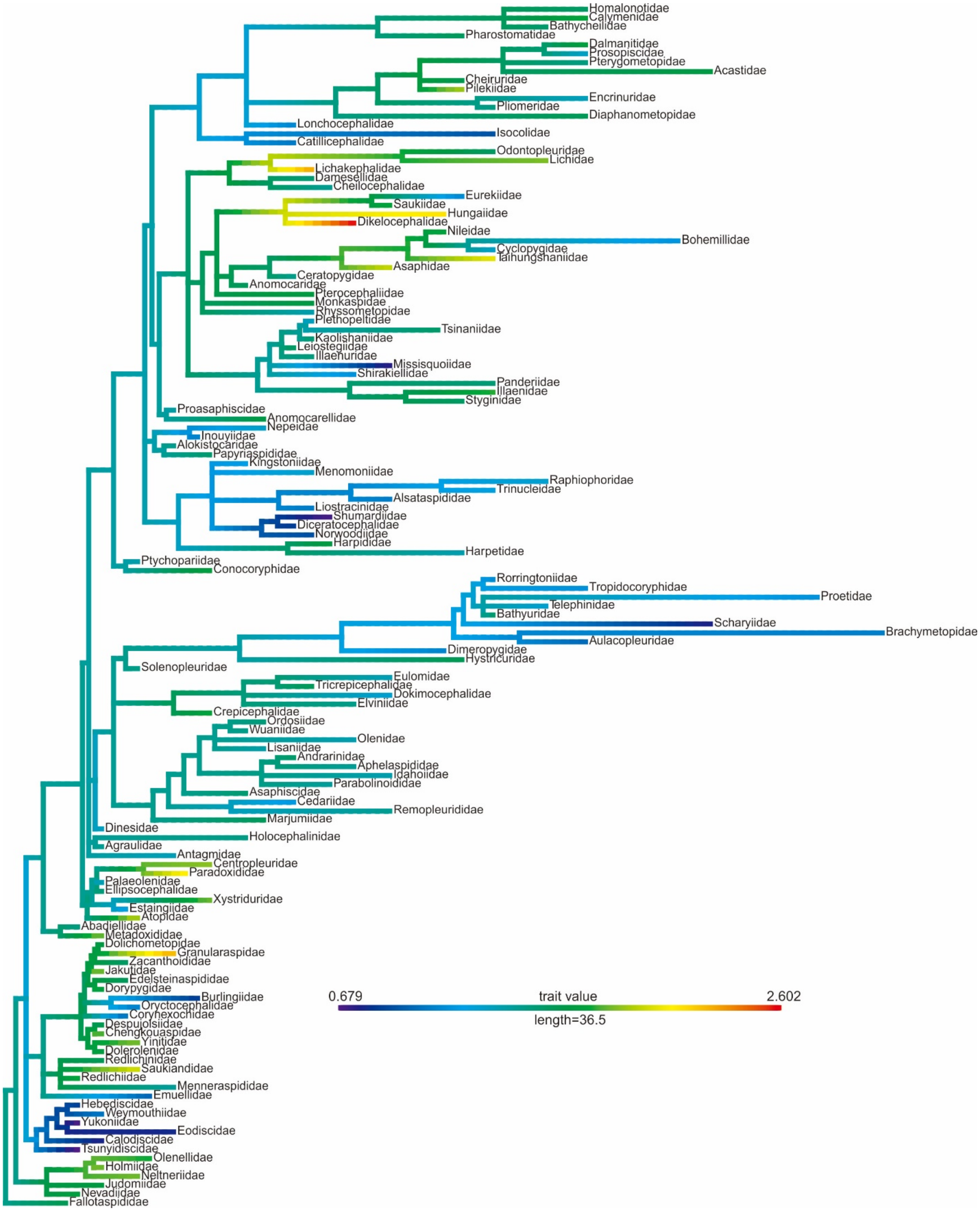

Fig. S10. Ancestral state reconstruction of maximum body size (mm) for major Cambrian and Ordovician trilobite families. Date from Datasets S3, 4, 6.

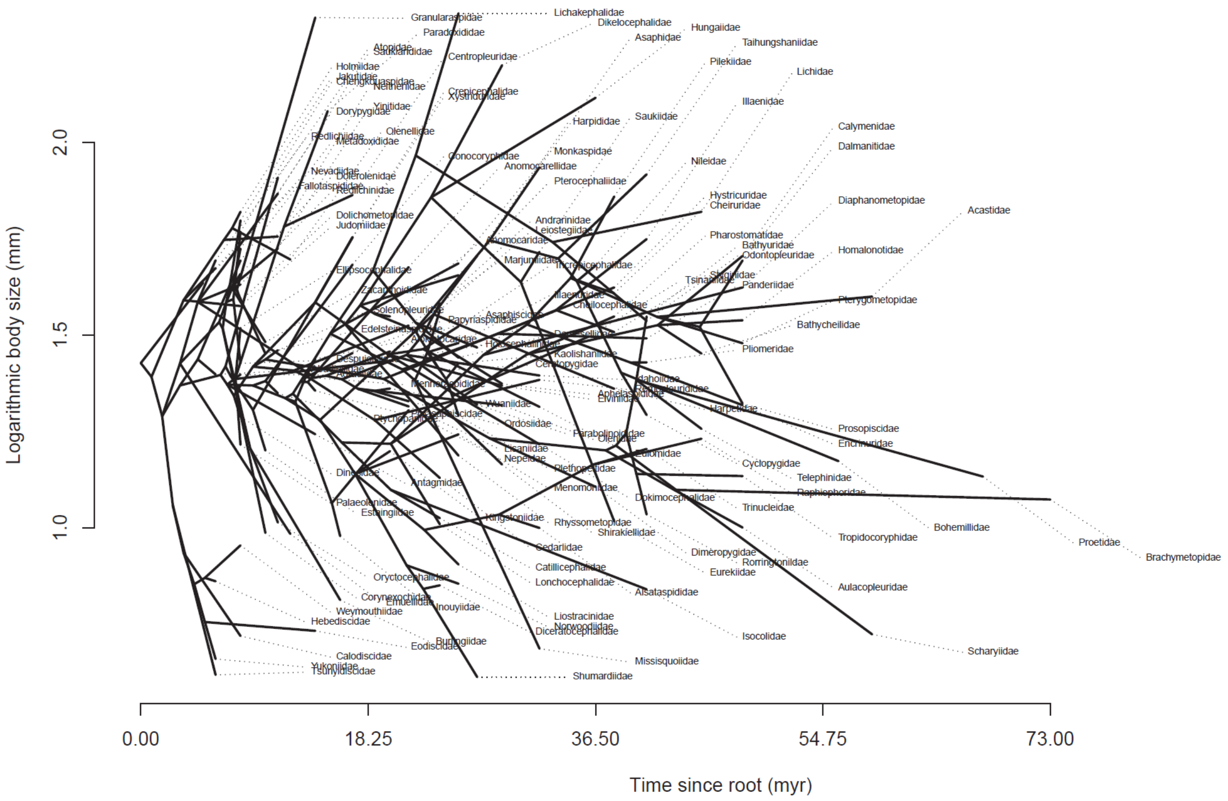

Fig. S11. Traitgram of mean body size (mm) for major Cambrian and Ordovician trilobite families. Date from Datasets S3-5.

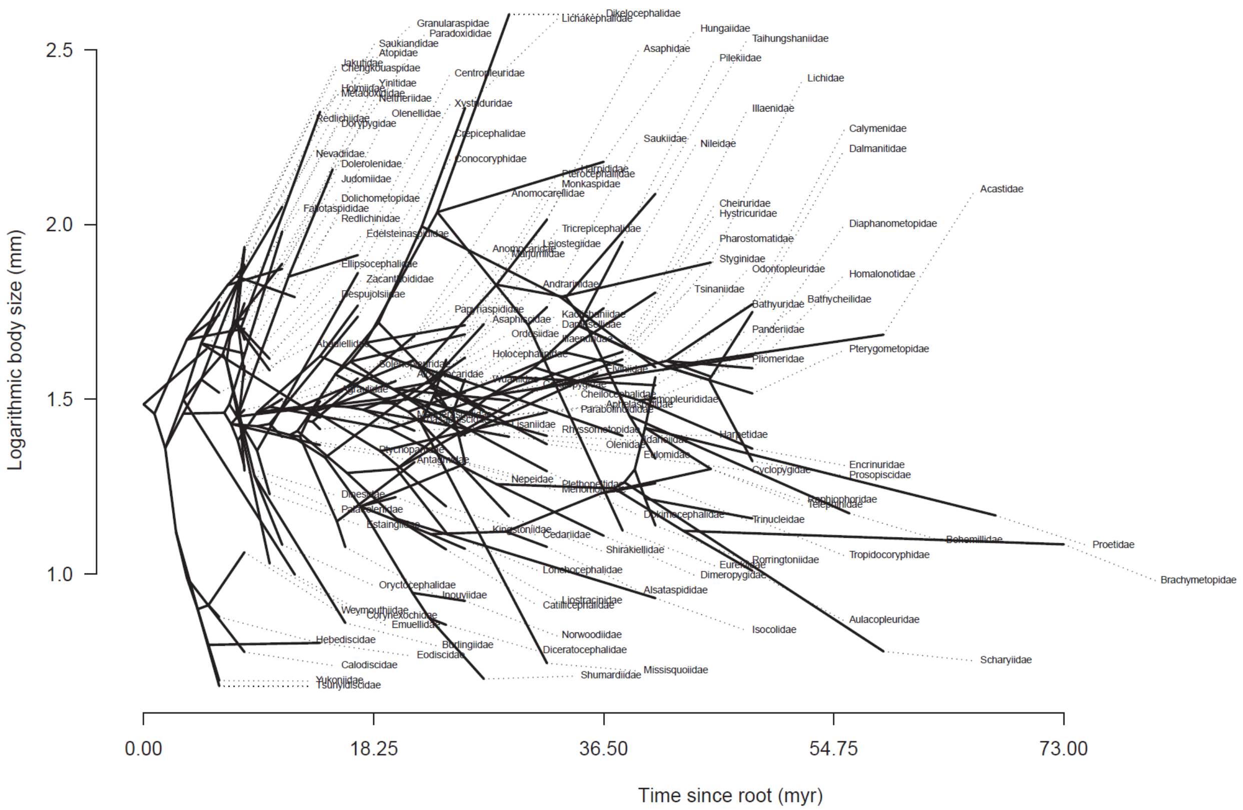

Fig. S12. Traitgram of maximum body size (mm) for major Cambrian and Ordovician trilobite families. Date from Datasets S4, 4, 6.

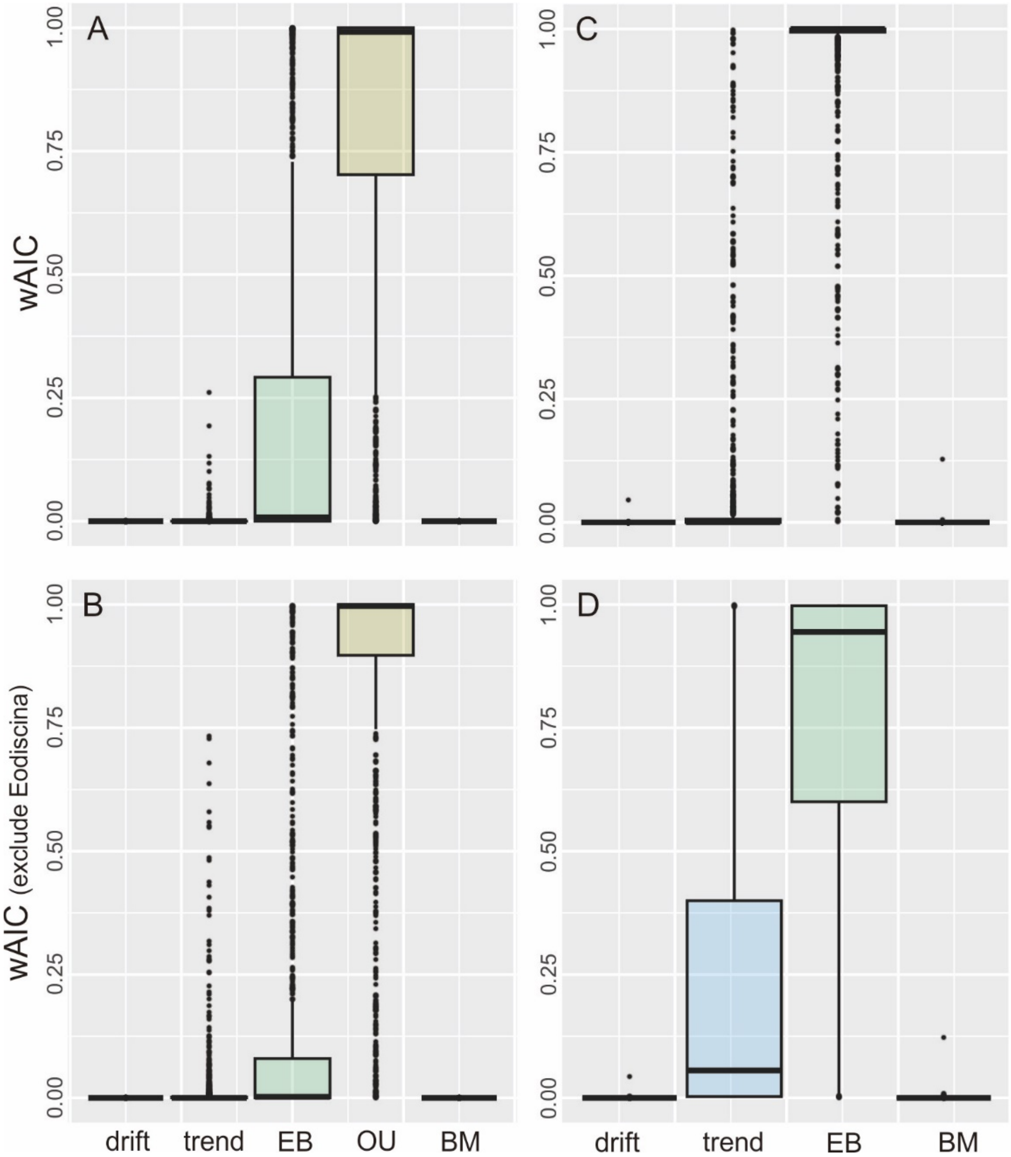

Fig. S13. Model-fitting results for trilobites using Akaike's information criterion weights (wAIC) for different models: drift, trend, Early Brust (EB), Ornstein–Uhlenbeck (OU), and Brownian motion (BM). Considering the phylogenetic affinity of the Eodiscina is unresolved (caption of fig. 8), the same analysis was performed on the body size dataset with Eodiscina removed (B). The OU model consistently got the largest wAIC (A, B), indicating more support than other models. However, the evolutionary processes that assumes by this model is rapid and commonly occur in generations level. There isn't expectation that this dynamic should be biologically meaningful on our ~3 Myr time scale. Therefore, we consider the results after removing the OU model (C, D). When the OU model is not considered, EB becomes the most supported model, while the other models are hardly supported. Date from Datasets S8 (A), S10 (B), S12 (C), S14 (D).

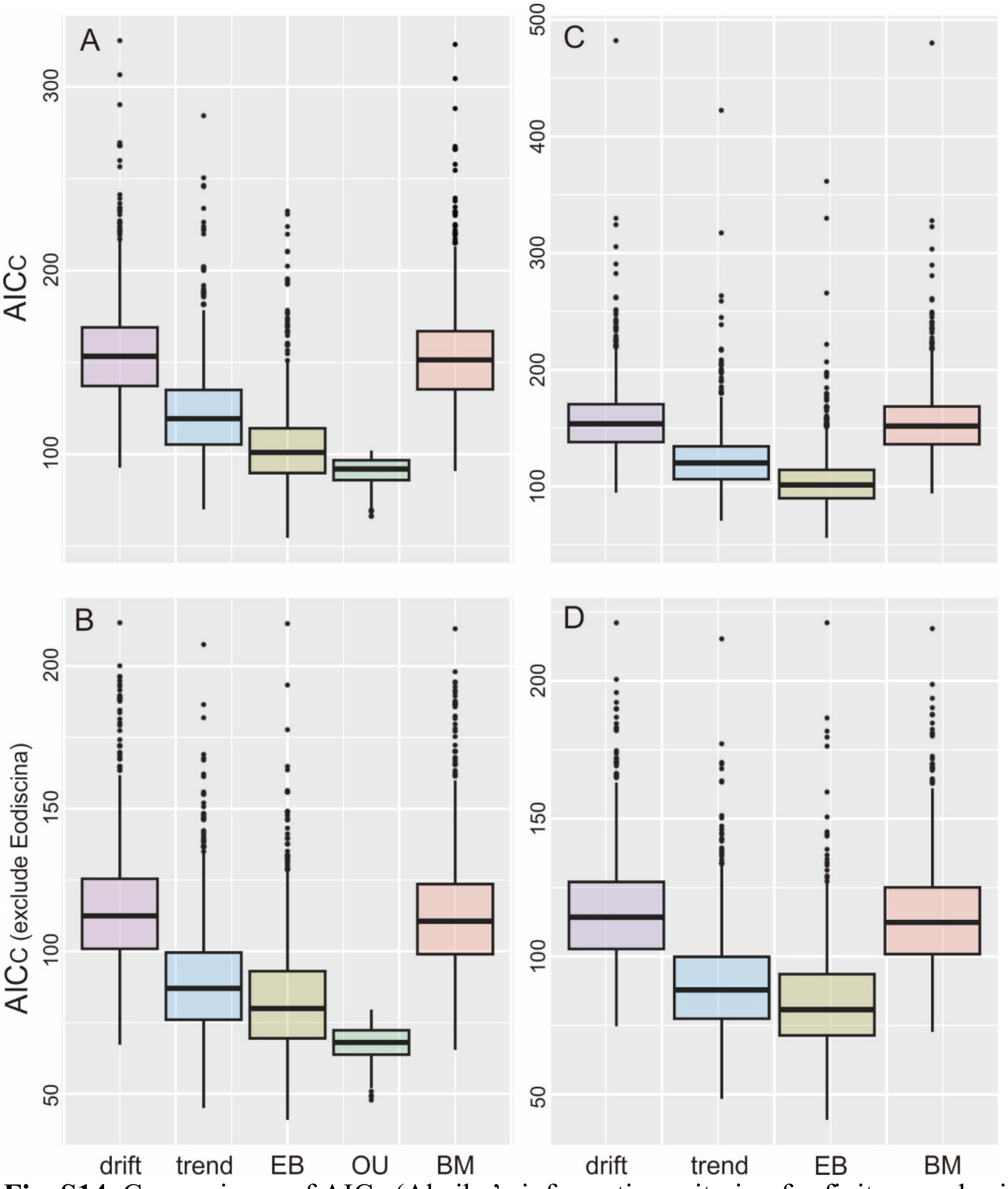

Fig. S14. Comparisons of AICc (Akaike's information criterion for finite sample sizes) values for drift, trend, Early Brust (EB), Ornstein–Uhlenbeck (OU), and Brownian motion (BM) models. Data greater than 350 in figure (A) are not shown. Lowest AICc scores indicate that the model fits the data better than any other tested model. The analyses shown in figs. B, D remove Eodiscina, suggesting no distinct difference when compared to the origin dataset. Figs. C, D represents the analysis results after excluding OU model, shows the EB model to be the most supported model. Date from Datasets S9 (A), S11 (B), S13 (C), S15 (D).

Additional supplementary datasets associated with this article:

Dataset S1: The dataset of 4,732 complete Cambrian and Ordovician trilobite specimens, including their body size value, classifications at the species, genus, family, and order levels, maximum and minimum chronostratigraphic ages, occurrences, and references.

Dataset S2: Time scale framework used in this work (see Materials and Methods).

Dataset S3: Informal supertree of Cambrian and Ordovician trilobites, containing 136 trilobite families. The tree file (.tre) can be opened by FigTree, Mesquite or other applicable software.

Dataset S4: The geological range information of trilobite families in our supertree, expressed as first and last appearance dates (FAD and LAD) in the fossil record. Range data is used to time-calibrate the phylogenetic tree by 'timePaleoPhy' function in the 'paleotree' R package (69).

Dataset S5: Mean body size (mm) for Cambrian and Ordovician trilobite families in supertree, comes from the logarithmic average of the mean value of all individuals in each family.

Dataset S6: Maximum body size (mm) for Cambrian and Ordovician trilobite families in supertree, comes from the logarithmic average of the maximum value of all individuals in each family.

Dataset S7: The R code used for the ancestral state reconstruction and model-fitting analysis in this article.

Dataset S8, 9: Akaike's information criterion weights (wAIC) and Akaike's information criterion for finite sample sizes (AICc) for five different models [drift, trend, Early Brust (EB), Ornstein–Uhlenbeck (OU), and Brownian motion (BM)] each with 1000 iterations.

Dataset S10, 11: Akaike's information criterion weights (wAIC) and Akaike's information criterion for finite sample sizes (AICc) for five different models [drift, trend, Early Brust (EB), Ornstein–Uhlenbeck (OU), and Brownian motion (BM)] each with 1000 iterations. Eodiscina is not included in the database.

Dataset S12, 13: Akaike's information criterion weights (wAIC) and Akaike's information criterion for finite sample sizes (AICc) for four different models [drift, trend, Early Brust (EB), and Brownian motion (BM)] each with 1000 iterations.

Dataset S14, 15: Akaike's information criterion weights (wAIC) and Akaike's information criterion for finite sample sizes (AICc) for four different models [drift, trend, Early Brust (EB), and Brownian motion (BM)] each with 1000 iterations. Eodiscina is not included in the database.

Dataset S16: Environmental data used in this work, including carbonate I/Ca values from (28) and (41), and temperature value from bulk-rock and skeletal material δ18O data [from (75) and (76) respectively].

REFERENCES AND NOTES

1. D. Jablonski, Micro-and macroevolution: Scale and hierarchy in evolutionary biology and paleobiology. *Paleobiology* **26**, 15–52 (2000).

2. B. Deline, J. M. Greenwood, J. W. Clark, M. N. Puttick, K. J. Peterson, P. C. J. Donoghue, Evolution of metazoan morphological disparity. *Proc. Natl. Acad. Sci. U.S.A.* **115**, E8909–E8918 (2018).

3. J.-X. Fan, S.-Z. Shen, D. H. Erwin, P. M. Sadler, N. M. Leod, Q.-M. Cheng, X.-D. Hou, J. Yang, X.-D. Wang, Y. Wang, H. Zhang, X. Chen, G.-X. Li, Y.-C. Zhang, Y.-K. Shi, D.-X. Yuan, Q. Chen, L.-N. Zhang, C. Li, Y.-Y. Zhao, A high-resolution summary of Cambrian to early Triassic marine invertebrate biodiversity. *Science* **367**, 272–277 (2020).

4. M. J. Benton, P. C. J. Donoghue, Paleontological evidence to date the tree of life. *Mol. Biol. Evol.* **24**, 26–53 (2007).

5. R. H. Peters, *The Ecological Implications of Body Size* (Cambridge Univ. Press, 1983).

6. G. B. West, J. H. Brown, B. J. Enquist, A general model for the origin of allometric scaling laws in biology. *Science* **276**, 122–126 (1997).

7. J. H. Brown, J. F. Gillooly, A. P. Allen, V. M. Savage, G. B. West, Toward a metabolic theory of ecology. *Ecology* **85**, 1771–1789 (2004).

8. P. M. Sander, E. M. Griebeler, N. Klein, J. V. Juarbe, T. Wintrich, L. J. Revell, L. Schmitz, Early giant reveals faster evolution of large body size in ichthyosaurs than in cetaceans. *Science* **374**, eabf5787 (2021).

9. O. Sanisidro, M. C. Mihlbachler, J. L. Cantalapiedra, A macroevolutionary pathway to megaherbivory. *Science* **380**, 616–618 (2023).

10. R. B. J. Benson, N. E. Campione, M. T. Carrano, P. D. Mannion, C. Sullivan, P. Upchurch, D. C. Evans, Rates of dinosaur body mass evolution indicate 170 million years of sustained ecological innovation on the avian stem lineage. *PLOS Biol.* **12**, e1001853 (2014).

11. G. Burin, T. Park, T. D. James, G. J. Slater, N. Cooper, The dynamic adaptive landscape of cetacean body size. *Curr. Biol.* **33**, 1787–1794.e3 (2023).

12. E. D. Cope, *The Primary Factors of Organic Evolution* (Open Court Publishing Company, 1904).

13. N. A. Heim, M. L. Knope, E. K. Schaal, S. C. Wang, J. L. Payne, Cope's rule in the evolution of marine animals. *Science* **347**, 867–870 (2015).

14. C. Bergmann, Ueber die Verhältnisse der Wärmeökonomie der Thierezuihrer Grösse. *Gottinger Studien.* **3**, 595–708 (1847).

15. J. Alroy, Cope's rule and the dynamics of body mass evolution in North American fossil mammals. *Science* **280**, 731–734 (1998).

16. J. L. Payne, A. G. Boyer, J. H. Brown, S. Finnegan, M. Kowalewski, R. A. Krause Jr., S. Kathleen Lyons, C. R. McClain, D. W. McShea, P. M. Novack-Gottshall, F. A. Smith, J. A. Stempien, S. C. Wang, Two-phase increase in the maximum size of life over 3.5 billion years reflects biological innovation and environmental opportunity. *Proc. Natl. Acad. Sci. U.S.A.* **106**, 24–27 (2009).

17. G. Hunt, K. Roy, Climate change, body size evolution, and Cope's Rule in deep-sea ostracodes. *Proc. Natl. Acad. Sci. U.S.A.* **103**, 1347–1352 (2006).

18. J. L. Payne, C. R. McClain, A. G. Boyer, J. H. Brown, S. Finnegan, M. Kowalewski, R. A. Krause Jr., S. Kathleen Lyons, D. W. McShea, P. M. Novack-Gottshall, F. A. Smith, P. Spaeth, J. A. Stempien, S. C. Wang, The evolutionary consequences of oxygenic photosynthesis: A body size perspective. *Photosynth. Res.* **107**, 37–57 (2011).

19. W. C. E. P. Verberk, D. Atkinson, K. N. Hoefnagel, A. G. Hirst, C. R. Horne, H. Siepel, Shrinking body sizes in response to warming: Explanations for the temperature-size rule with special emphasis on the role of oxygen. *Biol. Rev. Camb. Philos. Soc.* **96**, 247–268 (2021).

20. Z. Zhang, M. Augustin, J. L. Payne, Phanerozoic trends in brachiopod body size from synoptic data. *Paleobiology* **41**, 491–501 (2015).

21. M. E. Clapham, J. A. Karr, Environmental and biotic controls on the evolutionary history of insect body size. *Proc. Natl. Acad. Sci. U.S.A.* **109**, 10927–10930 (2012).

22. J. R. Paterson, G. D. Edgecombe, M. S. Y. Lee, Trilobite evolutionary rates constrain the duration of the Cambrian explosion. *Proc. Natl. Acad. Sci. U.S.A.* **116**, 4394–4399 (2019).

23. M. Foote, Contributions of individual taxa to overall morphological disparity. *Paleobiology* **19**, 403–419 (1993).

24. M. J. Hopkins, R. To, Long-term clade-wide shifts in trilobite segment number and allocation during the Palaeozoic. *Proc. R. Soc. London Ser. B* **289**, 20221765 (2022).

25. B. C. Gill, T. W. Lyons, S. A. Young, L. R. Kump, A. H. Knoll, M. R. Saltzman, Geochemical evidence for widespread euxinia in the Later Cambrian ocean. *Nature* **469**, 80–83 (2011).

26. C. T. Edwards, M. R. Saltzman, D. L. Royer, D. A. Fike, Oxygenation as a driver of the Great Ordovician Biodiversification Event. *Nat. Geosci.* **10**, 925–929 (2017).

27. T.-C. He, M.-Y. Zhu, B. J. W. Mills, P. M. Wynn, A. Y. Zhuravlev, R. Tostevin, P. A. E. Pogge von Strandmann, A. Yang, S. W. Poulton, G. A. Shields, Possible links between extreme oxygen perturbations and the Cambrian radiation of animals. *Nat. Geosci.* **12**, 468–474 (2019).

28. A. Lindskog, S. A. Young, C. N. Bowman, N. P. Kozik, S. M. Newby, M. E. Eriksson, J. Pettersson, E. Molin, J. D. Owens, Oxygenation of the Baltoscandian shelf linked to Ordovician biodiversification. *Nat. Geosci.* **16**, 1047–1053 (2023).

29. J. C. Gutiérrez-Marco, A. A. Sá, D. C. García-Bellido, I. Rábano, M. Valério, Giant trilobites and trilobite clusters from the Ordovician of Portugal. *Geology* **37**, 443–446 (2009).

30. D. M. Rudkin, G. A. Young, R. J. Elias, E. P. Dobrzanski, The world's biggest trilobite—*Isotelus rex* new species from the Upper Ordovician of northern Manitoba, Canada. *J. Paleontol.* **77**, 99–112 (2003).

31. M. A. Bell, S. J. Braddy, Cope's rule in the Ordovician trilobite family Asaphidae (order Asaphida): Patterns across multiple most parsimonious trees. *Hist. Biol.* **24**, 223–230 (2012).

32. P. A. Jell, J. M. Adrain, Available generic names for trilobites. *Mem. Queensl. Mus.* **48**, 331–553 (2003).

33. M. A. Butler, A. A. King, Phylogenetic comparative analysis: A modeling approach for adaptive evolution. *Am. Nat.* **164**, 683–695 (2004).

34. L. J. Harmon, J. B. Losos, T. Jonathan Davies, R. G. Gillespie, J. L. Gittleman, W. Bryan Jennings, K. H. Kozak, M. A. McPeek, F. Moreno-Roark, T. J. Near, A. Purvis, R. E. Ricklefs, D. Schluter, J. A. Schulte II, O. Seehausen, B. L. Sidlauskas, O. Torres-Carvajal, T. W. Jason, A. Ø. Mooers, Early bursts of body size and shape evolution are rare in comparative data. *Evolution* **64**, 2385–2396 (2010).

35. G. Hunt, M. T. Carrano, Models and methods for analyzing phenotypic evolution in lineages and clades. *Paleontol. Soci. Pap.* **16**, 245–269 (2010).

36. X. Chen, H.-F. Ling, D. Vance, G. A. Shields-Zhou, M.-Y. Zhu, S. W. Poulton, L. M. Och, S.-Y. Jiang, D. Li, C. Archer, Rise to modern levels of ocean oxygenation coincided with the Cambrian radiation of animals. *Nat. Commun.* **6**, 7142 (2015).

37. D. Wang, H.-F. Ling, U. Struck, X.-K. Zhu, M.-Y. Zhu, T.-C. He, B. Yang, A. Gamper, G. A. Shields, Coupling of ocean redox and animal evolution during the Ediacaran-Cambrian transition. *Nat. Commun.* **9**, 2575 (2018).

38. A. Y. Zhuravlev, R. A. Wood, Anoxia as the cause of the mid-early Cambrian (Botomian) extinction event. *Geology* **24**, 311–314 (1996).

39. M. L. Hough, G. A. Shields, L. Z. Evins, H. Strauss, R. A. Henderson, S. Mackenzie, A major sulphur isotope event at c. 510 Ma: A possible anoxia–Extinction–Volcanism connection during the Early–Middle Cambrian transition? *Terra Nova* **18**, 257–263 (2006).

40. C. Chang, W.-X. Hu, K.-J. Huang, Z.-F. Wang, X.-L. Zhang, Mass extinction coincided with expanded continental margin euxinia during the Cambrian Age 4. *Geophys. Res. Lett.* **50**, e2023GL105560 (2023).

41. W. Lu, A. Ridgwell, E. Thomas, D. S. Hardisty, G.-M. Luo, T. J. Algeo, M. R. Saltzman, B. C. Gill, Y. Shen, H.-F. Ling, C. T. Edwards, M. T. Whalen, X. Zhou, K. M. Gutchess, L. Jin, R. E. M. Rickaby, H. C. Jenkyns, T. W. Lyons, T. M. Lenton, L. R. Kump, Z. Lu, Late inception of a resiliently oxygenated upper ocean. *Science* **361**, 174–177 (2018).

42. M. R. Saltzman, C. T. Edwards, J. M. Adrain, S. R. Westrop, Persistent oceanic anoxia and elevated extinction rates separate the Cambrian and Ordovician radiations. *Geology* **43**, 807–810 (2015).

43. C. T. Edwards, D. A. Fike, M. R. Saltzman, W. Lu, Z. Lu, Evidence for local and global redox conditions at an Early Ordovician (Tremadocian) mass extinction. *Earth Planet. Sci. Lett.* **481**, 125–135 (2018).

44. M. Liu, D.-Z. Chen, L. Jiang, R. G. Stockey, D. Aseal, B. Zhang, K. Liu, X.-R. Yang, D.-T. Yan, N. J. Planavsky, Oceanic anoxia and extinction in the latest Ordovician. *Earth Planet. Sci. Lett.* **588**, 117553 (2022).

45. C. M. Ø. Rasmussen, T. R. A. Vandenbroucke, D. Nogues-Bravo, S. Finnegan, Was the Late Ordovician mass extinction truly exceptional? *Trends Ecol. Evol.* **38**, 812–821 (2023).

46. X. Lu, G. J. Gilleaudeau, B. Kendall, Uranium isotopes in non-euxinic shale and carbonate reveal dynamic Katian marine redox conditions accompanying a decrease in biodiversity prior to the Late Ordovician Mass Extinction. *Geochim. Cosmochim. Acta* **364**, 22–43 (2024).

47. J. A. Trotter, I. S. Williams, C. R. Barnes, C. Lécuyer, R. S. Nicoll, Did cooling oceans trigger Ordovician biodiversification? Evidence from conodont thermometry. *Science* **321**, 550–554 (2008).

48. A. L. Stigall, C. T. Edwards, R. L. Freeman, C. M. Ø. Rasmussen, Coordinated biotic and abiotic change during the Great Ordovician Biodiversification Event: Darriwilian assembly of early Paleozoic building blocks. *Palaeogeogr. Palaeoclimatol. Palaeoecol.* **530**, 249–270 (2019).

49. G. Chapelle, L. S. Peck, Polar gigantism dictated by oxygen availability. *Nature* **399**, 114–115 (1999).

50. B. C. Gill, T. W. Dahl, E. U. Hammarlund, M. A. LeRoy, G. W. Gordon, D. E. Canfield, A. D. Anbar, T. W. Lyons, Redox dynamics of later Cambrian oceans. *Palaeogeogr. Palaeoclimatol. Palaeoecol.* **581**, 110623 (2021).

51. R. A. Fortey, R. M. Owens, Feeding habits in trilobites. *Palaeontology* **42**, 429–465 (1999).

52. G. Hunt, S. A. Wicaksono, J. E. Brown, K. G. MacLeod, Climate-driven body-size trends in the ostracod fauna of the deep Indian Ocean. *Palaeontology* **53**, 1255–1268 (2010).

53. J. Clavel, H. Morlon, Accelerated body size evolution during cold climatic periods in the Cenozoic. *Proc. Natl. Acad. Sci. U.S.A.* **114**, 4183–4188 (2017).

54. B. J. W. Mills, A. J. Krause, I. Jarvis, B. D. Cramer, Evolution of atmospheric O_2 through the phanerozoic, revisited, revisited. *Annu. Rev. Earth Planet. Sci.* **51**, 253–276 (2023).

55. D. M. Raup, Biological extinction in Earth history. *Science* **231**, 1528–1533 (1986).

56. J. D. Olden, Z. S. Hogan, M. J. Vander Zanden, Small fish, big fish, red fish, blue fish: Size-biased extinction risk of the world's freshwater and marine fishes. *Glob. Ecol. Biogeogr.* **16**, 694–701 (2007).

57. P. M. Monarrez, N. A. Heim, J. L. Payne, Mass extinctions alter extinction and origination dynamics with respect to body size. *Proc. R. Soc. London Ser. B Biol. Sci.* **288**, 20211681 (2021).

58. P. M. Monarrez, N. A. Heim, J. L. Payne, Reduced strength and increased variability of extinction selectivity during mass extinctions. *R. Soc. Open Sci.* **10**, 230795 (2023).

59. F. A. Smith, R. E. E. Smith, S. K. Lyons, J. L. Payne, Body size downgrading of mammals over the late Quaternary. *Science* **360**, 310–313 (2018).

60. J. L. Payne, N. A. Heim, Body size, sampling completeness, and extinction risk in the marine fossil record. *Paleobiology* **46**, 23–40 (2020).

61. J. L. Payne, Evolutionary dynamics of gastropod size across the end-Permian extinction and through the Triassic recovery interval. *Paleobiology* **31**, 269–290 (2005).

62. P. M. Hull, S. A. F. Darroch, D. H. Erwin, Rarity in mass extinctions and the future of ecosystems. *Nature* **528**, 345–351 (2015).

63. N. C. Hughes, A. Minelli, G. Fusco, The ontogeny of trilobite segmentation: A comparative approach. *Paleobiology* **32**, 602–627 (2006).

64. S.-C. Peng, L. E. Babcock, P. Ahlberg, "The Cambrian Period" in *Geologic Time Scale 2020, Volume 2* (Elsevier, 2020).

65. D. Goldman, P. M. Sadler, S. A. Leslie, M. J. Melchin, F. P. Agterberg, F. M. Gradstein, "The Ordovician Period" in *Geologic Time Scale 2020, Volume 2* (Elsevier, 2020).

66. L. Babcock, S.-C. Peng, P. Ahlberg, X. Zhang, M.-Y. Zhu, P. Yu. Parkhaev, A model for subdividing Cambrian stages into substages. Abstract book, in *3rd International Congress on Stratigraphy* (Società Geologica Italiana, 2019), p. 142.

67. G. Geyer, A comprehensive Cambrian correlation chart. *Episodes* **42**, 321–332 (2019).

68. B. S. Lieberman, T. S. Karim, Tracing the trilobite tree from the root to the tips: A model marriage of fossils and phylogeny. *Arthropod Struct. Dev.* **39**, 111–123 (2010).

69. D. W. Bapst, paleotree: An R package for paleontological and phylogenetic analyses of evolution. *Methods Ecol. Evol.* **3**, 803–807 (2012).

70. E. Paradis, *Analysis of Phylogenetics and Evolution with R* (Springer Science & Business Media, 2011).

71. L. J. Revell, phytools: An R package for phylogenetic comparative biology (and other things). *Methods Ecol. Evol.* **3**, 217–223 (2012).

72. L. J. Harmon, J. T. Weir, C. D. Brock, R. E. Glor, W. Challenger, GEIGER: Investigating evolutionary radiations. *Bioinformatics* **24**, 129–131 (2008).

73. J. Felsenstein, Maximum-likelihood estimation of evolutionary trees from continuous characters. *Am. J. Hum. Genet.* **25**, 471–492 (1973).

74. T. H. Torsvik, L. R. M. Cocks, "Cambrian" in *Earth History and Palaeogeography*, T. H. Torsvik, L. R. M. Cocks, Eds. (Cambridge Univ. Press, 2017).

75. S. L. Goldberg, T. M. Present, S. Finnegan, K. D. Bergmann, A high-resolution record of early Paleozoic climate. *Proc. Natl. Acad. Sci. U.S.A.* **118**, e2013083118 (2021).

76. C. R. Scotese, H.-J. Song, B. J. W. Mills, D. G. van der Meer, Phanerozoic paleotemperatures: The earth's changing climate during the last 540 million years. *Earth Sci. Rev.* **215**, 103503 (2021).

77. D. A. Harper, B. Cascales-Miñana, T. Servais, Early Palaeozoic diversifications and extinctions in the marine biosphere: A continuum of change. *Geol. Mag.* **157**, 5–21 (2020).

78. F. A. Sundberg, G. Geyer, P. D. Kruse, L. B. McCollum, T. V. Pegel, A. Zylinska, A. Y. Zhuravlev, International correlation of the Cambrian Series 2-3, Stages 4-5 boundary interval. *Australas. Palaeontol. Mem.* **49**, 83–124 (2016).

79. A. Bignon, B. G. Waisfeld, N. E. Vaccari, B. D. Chatterton, Reassessment of the Order Trinucleida (Trilobita). *J. System. Palaeontol.* **18**, 1061–1077 (2020).

80. N. C. Hughes, Ontogeny, intraspecific variation, and systematics of the Late Cambrian trilobite *Dikelocephalus*. *Smithson. Contrib. Paleobiol.* **79**, 1–89 (1994).

81. R. A. Fortey, The first known complete lichakephalid trilobite, Lower Ordovician of Morocco. *Mem. Assoc. Australas. Palaeontol.* **42**, 1–7 (2012).

82. J. M. Adrain, Class Trilobita Walch, 1771. *Zootaxa* **3148**, 104–109 (2011).

83. P. A. Jell, Phylogeny of early cambrian trilobites. *Spec. Pap. Palaeontol.* **70**, 45–57 (2003).

84. S. Lee, D. Lee, D. K. Choi, Cambrian–Ordovician trilobite family Missisquoiidae Hupé, 1955: Systematic revision and palaeogeographical considerations based on cladistic analysis. *Palaeogeogr. Palaeoclimatol. Palaeoecol.* **260**, 315–341 (2008).

85. T. S. Park, J. E. Kim, S. Lee, D. K. Choi, *Mansuyia* Sun, and *Tsinania* Walcott, from the Furongian of North China and the evolution of the trilobite family Tsinaniidae. *Palaeontology* **57**, 269–282 (2014).

86. R. A. Fortey, R. M. Owens, The Arenig series in South Wales. Bulletin of the British Museum (Natural History). *Geology* **41**, 69–305 (1987).

87. B. S. Lieberman, Phylogenetic analysis of some basal Early Cambrian Trilobites, The Biogeographic origins of the Eutrilobita, and the timing of the Cambrian Radiation. *J. Paleontol.* **76**, 692–708 (2002).

88. J. R. Paterson, G. D. Edgecombe, The Early Cambrian Trilobite Family Emuellidae Pocock, 1970: Systematic position and revision of Australian species. *J. Paleontol.* **80**, 496–513 (2006).

89. A. R. Palmer, L. N. Repina, Through a glass darkly: Taxonomy, phylogeny, and biostratigraphy of the Olenellina. *Univ. Kansas Paleontol. Contrib.* **3**, 1–35 (1993).

90. P. A. Jell, "Introduction to the suborder Eodiscina" in *Treatise on Invertebrate Paleontology, Part O, Arthropoda 1. Trilobita, Revised. Volume 1* R. L. Kaesler, Ed. (Geological Society of America and Univ. of Kansas Press, 1997).

91. W.-T. Zhang, Y.-H. Lu, Z.-L. Zhu, Y.-Y. Qian, H.-L. Lin, Z.-Y. Zhou, S.-G. Zhang, J.-L. Yuan, *Cambrian trilobite faunas of southwestern China* (Science Press, 1980).

92. N. P. Suvorova, Corynexochoid trilobites and their evolutionary history. *Trudy Paleontol. Inst.* **103**, 1–319 (1964).

93. J. O. Ebbestad, G. E. Budd, Burlingiid trilobites from Norway, with a discussion of their affinities and relationships. *Palaeontology* **45**, 1171–1195 (2002).

94. G. Geyer, Exotic trilobites from the Lower–Middle Cambrian boundary interval in Morocco and their bearing on the Cambrian Series 3 lower boundary. *Paläontol. Z.* **89**, 749–781 (2015).

95. N. P. Suvorova, Cambrian trilobites from the eastern Siberian Platform. Part 2. Olenellidae-Granulariidae. *Trudy Paleontol. Inst.* **84**, 1–238 (1960).

96. T. J. Cotton, The phylogeny and systematics of blind Cambrian ptychoparioid trilobites. *Palaeontology* **44**, 167–207 (2001).

97. L. E. Babcock, Systematics and phylogenetics of polymeroid trilobites from the Henson Gletscher and Kap Stanton Formations (Middle Cambrian), north Greenland. *Bull. Grønl. Geol. Unders.* **169**, 79–127 (1994).

98. J. C. Lamsdell, P. A. Selden, Phylogenetic support for the monophyly of proetide trilobites. *Lethaia* **48**, 375–386 (2015).

99. L. N. Repina, V. V. Khomentovsky, I. T. Zhuravleva, A. Yu. Rozanov, *Lower Cambrian Biostratigraphy of the Sayan-Altay Folded Region* (Akademiya Nauk SSSR, Sibirskoe Otdelenie, Institut Geologii i Geofiziki, Izdatelstvo, 1964).

100. J. M. Adrain, S. E. Peters, S. R. Westrop, The Marjuman trilobite *Cedarina* Lochman: Thoracic morphology, systematics, and new species from western Utah and eastern Nevada, USA. *Zootaxa* **2218**, 35–58 (2009).

101. D. S. Monti, V. A. Confalonieri, First cladistic analysis of the trilobite family Olenidae from the Furongian and Ordovician. *Lethaia* **52**, 304–322 (2019).

102. C. J. Bentley, J. B. Jago, Wuaniid trilobites of Australia. *Mem. Assoc. Australas. Palaeontol.* **30**, 179–191 (2004).

103. D. Lee, B. D. Chatterton, Protaspides of Upper Cambrian *Aphelaspis* (Ptychopariida, Trilobita) and related species with their taxonomic implications. *Palaeontology* **48**, 1351–1375 (2005).

104. D. Lee, B. D. Chatterton, Hystricurid trilobite larvae from the Garden City Formation (Lower Ordovician) of Idaho and their phylogenetic implications. *J. Paleontol.* **71**, 862–877 (1997).

105. J. M. Adrain, A synopsis of Ordovician trilobite distribution and diversity. *Geol. Soc. Lond. Mem.* **38**, 293–332 (2013).

106. M. C. Ebach, K. McNamara, A systematic revision of the family Harpetidae (Trilobita). *Rec. West. Aust. Mus.* **21**, 235–267 (2002).

107. J.-L. Yuan, W.-T. Zhang, Z.-L. Zhu, *Cambrian Stratigraphy and Trilobite Fauna in Southern and Western Marginal Parts of the Ordos Platform* (Science Press, 2016).

108. F. A. Sundberg, Redescription of *Alokistocare subcoronatum* (Hall and Whitfield, 1877), the type species of *Alokistocare*, and the status of Alokistocaridae Resser, 1939B (Ptychopariida: Trilobita, Middle Cambrian). *J. Paleontol.* **73**, 1126–1143 (1999).

109. R. A. Fortey, “Classification” in *Treatise on Invertebrate Paleontology, Part O, Arthropoda 1. Trilobita, Revised. Volume 1*, R. L. Kaesler, Ed. (Geological Society of America and Univ. of Kansas Press, 1997).

110. P. D. Lane, A. T. Thomas, A review of the trilobite Suborder Scutelluina. *Spec. Pap. Palaeontol.* **30**, 141–160 (1983).

111. G. D. Edgecombe, “Trilobite phylogeny and the Cambrian–Ordovician “Event”: Cladistic reappraisal” in *Extinction and Phylogeny*, M. J. Novacek, Q. D. Wheeler, Eds. (Columbia Univ. Press, 1992).

112. R. A. Fortey, B. D. E. Chatterton, Classification of the trilobite suborder Asaphina. *Palaeontology* **31**, 165–222 (1988).

113. C. Lochman, The evolution of some upper cambrian and lower ordovician trilobite families. *J. Paleontol.* **30**, 445–462 (1956).

114. S. R. Westrop, J. D. Eoff, T. Ng, A. A. Dengler, J. M. Adrain, Classification of the Late Cambrian (Steptoean) trilobite genera *Cheilocephalus* Berkey, 1898 and *Oligometopus* Resser, 1936 from Laurentia. *Can. J. Earth Sci.* **45**, 725–744 (2008).

115. R. A. Fortey, Ontogeny, hypostome attachment and trilobite classification. *Palaeontology* **33**, 529–576 (1990).

116. L. Ramsköld, Pattern and process in the evolution of the Odontopleuridae (Trilobita). The Selenopeltinae and Ceratocephalinae. *Trans. R. Soc. Edinb. Earth Sci.* **82**, 143–181 (1991).

117. J. R. Pollitt, R. A. Fortey, M. A. Wills, Systematics of the trilobite families Lichidae Hawle & Corda, 1847 and Lichakephalidae Tripp, 1957: The application of Bayesian inference to morphological data. *J. Syst. Palaeontol.* **3**, 225–241 (2005).

118. F. R. Rasetti, Phylogeny of the Cambrian trilobite family Catillicephalidae and the ontogeny of *Welleraspis*. *J. Paleontol.* **28**, 599–612 (1954).

119. R. A. Fortey, Cambrian-Ordovician trilobites from the boundary beds in western Newfoundland and their phylogenetic significance. *Spec. Pap. Palaeontol.* **30**, 179–211 (1983).

120. B. D. Chatterton, D. J. Siveter, G. D. Edgecombe, A. S. Hunt, Larvae and relationships of the Calymenina (Trilobita). *J. Paleontol.* **64**, 255–277 (1990).

121. R. A. Fortey, Trilobite systematics: The last 75 years. *J. Paleontol.* **75**, 1141–1151 (2001).

122. J. M. Adrain, S. R. Westrop, E. Landing, R. A. Fortey, Systematics of the Ordovician trilobites *Ischyrotoma* and *Dimeropygiella*, with species from the type Ibexian area, western U.S.A. *J. Paleontol.* **75**, 947–971 (2001).

123. J. Moysiuk, J. B. Caron, Burgess Shale fossils shed light on the agnostid problem. *Proc. R. Soc. London Ser. B* **286**, 20182314 (2019).
